# Efficient and Reproducible Myogenic Differentiation from Human iPS Cells: Prospects for Modeling Miyoshi Myopathy *In Vitro*

**DOI:** 10.1371/journal.pone.0061540

**Published:** 2013-04-23

**Authors:** Akihito Tanaka, Knut Woltjen, Katsuya Miyake, Akitsu Hotta, Makoto Ikeya, Takuya Yamamoto, Tokiko Nishino, Emi Shoji, Atsuko Sehara-Fujisawa, Yasuko Manabe, Nobuharu Fujii, Kazunori Hanaoka, Takumi Era, Satoshi Yamashita, Ken-ichi Isobe, En Kimura, Hidetoshi Sakurai

**Affiliations:** 1 Center for iPS Cell Research and Application (CiRA), Kyoto University, Sakyo-ku, Kyoto, Japan; 2 Department of Immunology, Nagoya University Graduate School of Medicine, Showa-ku, Nagoya, Japan; 3 Department of Histology and Cell Biology, School of Medicine, Kagawa University, Miki-cho, Kida-gun, Kagawa, Japan; 4 Department of Growth Regulation, Institute for Frontier Medical Sciences, Kyoto University, Sakyo-ku, Kyoto, Japan; 5 Department of Health Promotion Sciences, Graduate School of Human Health Sciences, Tokyo Metropolitan University, Hachioji City, Tokyo, Japan; 6 Molecular Embryology, Department of Bioscience, School of Science, Kitasato University, Minami-ku, Sagamihara City, Kanagawa, Japan; 7 Department of Cell Modulation, Institute of Molecular Embryology and Genetics, Kumamoto University, Chuo-ku, Kumamoto, Japan; 8 Department of Neurology, Graduate School of Medical Sciences, Kumamoto University, Chuo-ku, Kumamoto, Japan; University of Minnesota Medical School, United States of America

## Abstract

The establishment of human induced pluripotent stem cells (hiPSCs) has enabled the production of *in vitro*, patient-specific cell models of human disease. *In vitro* recreation of disease pathology from patient-derived hiPSCs depends on efficient differentiation protocols producing relevant adult cell types. However, myogenic differentiation of hiPSCs has faced obstacles, namely, low efficiency and/or poor reproducibility. Here, we report the rapid, efficient, and reproducible differentiation of hiPSCs into mature myocytes. We demonstrated that inducible expression of *myogenic differentiation1* (*MYOD1*) in immature hiPSCs for at least 5 days drives cells along the myogenic lineage, with efficiencies reaching 70–90%. Myogenic differentiation driven by *MYOD1* occurred even in immature, almost completely undifferentiated hiPSCs, without mesodermal transition. Myocytes induced in this manner reach maturity within 2 weeks of differentiation as assessed by marker gene expression and functional properties, including *in vitro* and *in vivo* cell fusion and twitching in response to electrical stimulation. Miyoshi Myopathy (MM) is a congenital distal myopathy caused by defective muscle membrane repair due to mutations in DYSFERLIN. Using our induced differentiation technique, we successfully recreated the pathological condition of MM *in vitro*, demonstrating defective membrane repair in hiPSC-derived myotubes from an MM patient and phenotypic rescue by expression of full-length *DYSFERLIN (DYSF)*. These findings not only facilitate the pathological investigation of MM, but could potentially be applied in modeling of other human muscular diseases by using patient-derived hiPSCs.

## Introduction

The establishment of human induced pluripotent stem cells (hiPSCs) [1], [2] has paved the way for the generation of patient-specific stem cell resources. Directed differentiation of pluripotent stem cells into a variety of cell types provides a powerful tool for *in vitro* disease modeling [3]. Although the number and genetic diversity of patient-derived hiPSC lines continues to increase, the difficulty of differentiating hiPSC into mature cell types remains a major obstacle in understanding disease.

Effective differentiation into affected cell types is a critical step in the production of *in vitro* disease models from hiPSCs. In the case of myopathies, significant efforts have been made to generate skeletal muscle cells from human pluripotent stem cells [4], [5], [6]. However, previously reported differentiation protocols suffer from complex time-consuming procedures, low differentiation efficiencies, and/or low reproducibility. Reproducibility is perhaps the greatest hurdle facing robust differentiation protocols from human pluripotent stem cells, especially considering the high levels of clonal variation previously reported [7].

Directed myogenic differentiation of adult somatic cells mediated by the master transcriptional factor, MYOD1 [8], [9], was initially established in 1987 [8]. Following this first demonstration, various types of cells have been shown to give rise to myocytes in response to forced expression of *MYOD1* [9], [10], [11], including hiPSC-derived fibroblasts treated with *MYOD1* mRNA [12]. Considering the inherent potential of hiPSCs, differentiation into fibroblasts prior to myogenic induction is a redundant step. Recently, Tedesco et al. showed that hiPSC-derived mesoangioblast-like stem/progenitor cells can be converted into myocytes by tamoxifen-induced MYOD-ER overexpression [13]. Goudenege et al. also showed that hiPSC-derived mesenchymal cells can be promoted to myogenic differentiation efficiently by Adenoviral-transduction mediated *MYOD1* overexpression [14]. The 2 reports both indicated that iPSC-derived mesodermal or mesenchymal cells, both of which are differentiated for more than 2 weeks from undifferentiated hiPSCs, have a high potential for myogenic differentiation in response to *MYOD1* overexpression. However, such differentiation steps prior to *MYOD1* transduction might contribute to the reported observation of low reproducibility. Because mouse embryonic stem cells (mESCs) are able to directly differentiate to myocytes in response to Tetracycline (Tet)-induced *MYOD1* expression [15], we assessed whether drug-induced *MYOD1* expression could similarly promote efficient myocyte differentiation directly from undifferentiated hiPSCs. Here, we demonstrate that *MYOD1* overexpression in immature hiPSCs drives them to mature as myocytes with very high efficiency and reproducibility within 2 weeks.

Miyoshi myopathy [16] (MM) is a congenital distal myopathy caused by defective muscle membrane repair as a result of mutations in DYSFERLIN [17], [18]. Research directed at understanding the MM pathology has been primarily performed using model mice. To evaluate the true pathology of human disease, it is important to exploit current iPSC technology for direct assessment of patient samples. Here, we apply our differentiation technique to MM-disease modeling, recapitulating disease pathology *in vitro*, and successfully rescue the phenotype of MM by gene repair. The differentiation method presented here is suitable for muscular disease modeling by using patient-derived hiPSC. Our method of MYOD1-induced myogenic differentiation directly from undifferentiated hiPSCs is also suitable for the establishment of drug screening systems by using patient-derived hiPSCs. This is due to the unlimited proliferation potential of hiPSCs and uniformity in the undifferentiated state, which may prevent variation in differentiation frequency.

## Results

### Generation of hiPSCs with Drug-inducible *MYOD1* Expression

**Figure 1:**
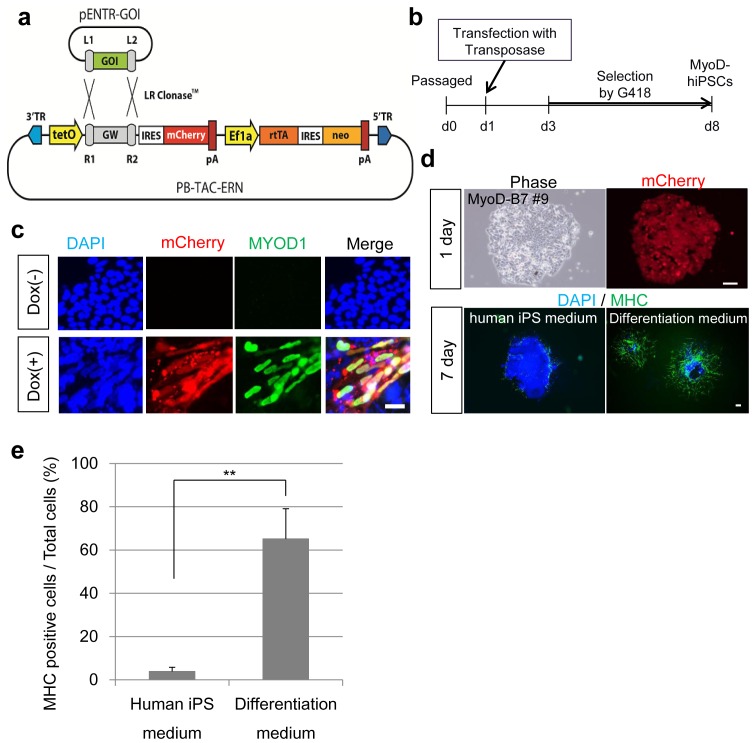
Generation of MyoD-hiPSCs. (**a**) Construction of the Tet-inducible *MYOD1* expressing *piggyBac* vector (Tet-MyoD1 vector). (**b**) A scheme of generation of MyoD-hiPSCs. Human iPSCs were transfected the Tet-MyoD1 vectors with transposase by lipofection. To select transfected cells, G418 were added for 5 days in the hiPSC culture media at 2 days after transfection. (**c**) MyoD-hiPSCs after 24 h in culture with or without Dox administration. Scale bar = 20 µm. (**d**) Upper lanes show dox-added MyoD-hiPSCs at d1. Lower lanes show immunodetection of MHC in Dox-induced MyoD-hiPSCs at d7. A lower-left panel shows the cells differentiated in maintenance medium from d1 to d7. A lower-right panel shows the cells differentiated in αMEM containing 5% KSR from d1 to d7. Scale bars = 200 µm. (**e**) Percentage of MHC positive cells per total cells following MyoD-induced differentiation. **p<0.01.

We constructed a self-contained, drug-inducible expression vector, based on the *piggyBac* (PB) transposon [19]. This vector constitutively expresses the neomycin (G418) resistance gene along with the rtTA transactivator element, which mediates doxycycline (Dox)-dependent activation of cDNA cassettes controlled by tetO promoter (PB-TAC-ERN; **Fig. 1a**). Activation of gene expression in response to Dox may be indirectly monitored by co-incident mCherry activation. Using Gateway cloning, we produced a derivative vector containing the human *MYOD1* gene (Tet-MyoD1). The Tet-MyoD1 vector was transfected together with PB transposase (PBase) into 3 independent hiPSC lines, and selected in G418 supplemented media for 5 days to generate pooled MyoD-hiPSCs containing genomic transposon integrations (**Fig. 1b**). In these pooled MyoD-hiPSCs, Dox administration for 24 h robustly induced *MYOD1* expression as detected by mCherry fluorescence and MYOD1 protein (**Fig. 1c**). As some cells did not express mCherry, we selected appropriate MyoD-hiPSC clones with robust, uniform levels of mCherry induction for further analysis. These clones retained pluripotency, which was confirmed by their expression of surface markers (**Fig. S1a**) and gene characteristic of an undifferentiated state (**Fig. S1b**), as well as teratoma formation (**Fig. S1c**). These clones were selected by the high expression of mCherry after Dox administration (**Fig. S2a**). RT-PCR analysis of these clones confirmed undetectable exogenous *MYOD1* background expression in the absence of Dox, whereas Dox treatment strongly induced exogenous *MYOD1* within 48 h, in correlation with mCherry expression (d2). Importantly, despite continued maintenance in hiPSC culture conditions, endogenous *MYOD1* activation was detectable after 96 h (d5) of Dox induction, with the effect being reproducible in MyoD-hiPSC clones derived from 3 distinct hiPSC lines: 201B7, 253G1, and 254G4 [1] (**Fig. S2b**). In selected MyoD-hiPSC clones, mCherry expression was detected uniformly 24 h after Dox administration in hiPSC-maintenance conditions (**Fig. 1d**). When the addition of Dox was continued for 7 days under hiPSC-maintenance conditions, the emergence of myosin heavy chain (MHC) [20] positive cells was extremely limited to the edge of MyoD-hiPSC colonies, and many MyoD-hiPSCs lines retaining undifferentiated colonies (**Fig. 1d**). On the other hand, when the culture medium was exchanged for differentiation medium on d1, MyoD-hiPSCs were differentiated into MHC positive myocytes disrupting colony formation. This medium replacement resulted in highly efficient myogenic induction (**Fig. 1e**). These results suggest that extended expression of transgenic *MYOD1* was able to activate the myogenic differentiation program in undifferentiated hiPSCs, even under sub-optimal conditions.

### Dox-induced Myogenic Differentiation of MyoD-hiPSCs

**Figure 2:**
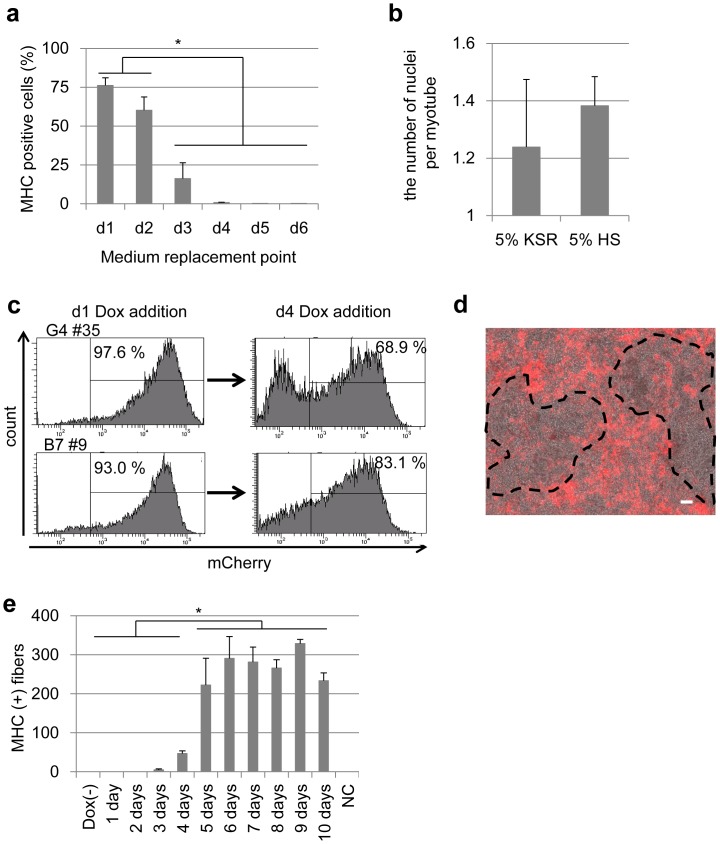
Optimization of Differentiation Conditions. (**a**) Percentage of MHC positive myogenic cells derived from MyoD-hiPSCs during 9 days differentiation with various timing of medium replacement. *p<0.05 (**b**) The average number of nuclei of myofibers in each condition of 5% KSR or 5% HS containing media after 7 days differentiation. (**c**) Flow cytometric analysis of MyoD-hiPSCs with 24 h Dox treatment in different start points. Dox addition at differentiation d1 promoted higher percentage of mCherry expression in MyoD-hiPSCs than Dox addition at differentiation d4. (**d**) A merged image of phase-contrast and mCherry images in differentiated MyoD-hiPSCs which were administrated Dox at differentiation d4. Some MyoD-hiPSCs turned to be unresponsive with Dox, indicating no mCherry expression area (dotted line). Scale bar = 100 µm. (**e**) MHC positive myogenic cell number derived from MyoD-hiPSCs during 11 days differentiation with various administration periods of Dox. *p<0.05.

As MyoD-hiPSCs maintained an undifferentiated status in hiPSC maintenance medium (**Fig. 1d**), we sought to optimize the differentiation protocol by adjusting culture conditions to be more appropriate for myogenic induction; first removing basic-fibroblast growth factor (b-FGF) for spontaneous differentiation, and then changing to alpha Minimal Essential Medium (αMEM) supplemented with a low concentration (5%) of knockout serum replacement (KSR). Dox treatment was maintained throughout the analysis. Early medium replacement to low-KSR αMEM enhanced myogenic differentiation, whereas late replacement did not improve differentiation compared to continued culture in hiPSC maintenance media without bFGF (**Fig. 2a**). Media containing a low concentration of horse serum has been shown to promote the maturation of myoblast into myotubes [9], [21]. To examine this later stage of differentiation, we assessed the effect of serum on the maturation of Dox-induced myogenic cells by counting the number of nuclei per myotube on d9. Although the difference was not statistically significant, a tendency towards higher numbers of nuclei was detected in hiPSCs cultured in 5% horse serum as compared to 5% KSR (**Fig. 2b**).

**Figure 3:**
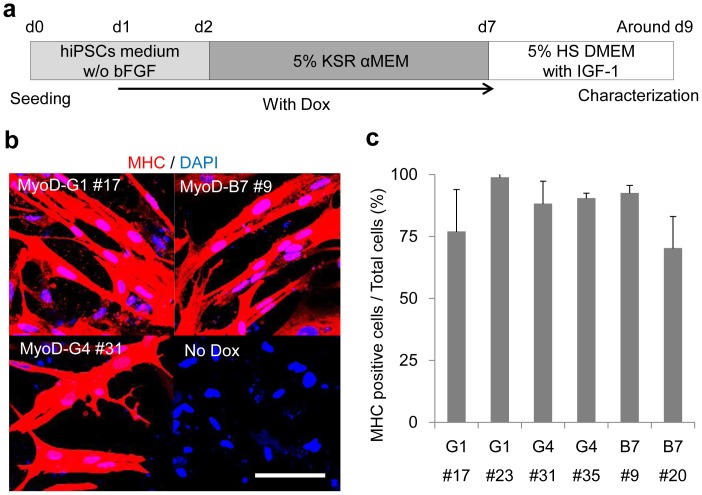
Reproducible myogenic differentiation with the optimized protocol. (**a**) A schematic of our muscle differentiation protocol beginning with MyoD-hiPSCs. (**b**) Immunohistochemistry of differentiated MyoD-hiPSCs for MHC (red). Scale bar = 100 µm. (**c**) Percentage of MHC positive cells per total cells following MyoD-induced differentiation of 6 MyoD-hiPSC clones. (n = 3 for each clone). Data are listed as mean ±S.D.

We predicted that the initiation point and period of Dox administration could also have an effect on differentiation responses. Dox addition at day 1 (d1) of differentiation (24 h after bFGF removal) induced over 90% mCherry positive cells. However, Dox addition later on in the differentiation protocol (d4) revealed an unresponsive, mCherry negative population (**Fig. 2c, d**), suggesting a failure of the expression vector to activate in a proportion of partially differentiated cells. To assess whether fully differentiated hiPSCs can be promoted into myogenic differentiation by Dox-mediated *MYOD1* expression, we applied our system to mesenchymal progenitor cells, known as SB-outgrowth cells (SB-OGs) [5], derived from hiPSCs. According to the report of Mahmood et al, SB-OGs derived from MyoD-hiPSCs were established after 14 days of differentiation, and were passaged twice [5]. When Dox was administrated to SB-OGs cultures, low levels of mCherry expression were observed, indicating low myogenic differentiation efficiency (**Fig. S3a, b**). Taken together, these results indicate that our inducible gene expression system using the *piggyBac* vector is suitable for driving the expression of genes in undifferentiated hiPSCs, rather than those which have been differentiated. However, Dox administration at the initiation of differentiation (d0; coincident with the change to low-serum media) led to lower cell survival (data not shown). Therefore, Dox administration was initiated at the fixed time point, d1, in our system. Next, we examined the period of Dox administration. MyoD-hiPSCs did not differentiate into myocytes following less than 3 days of Dox administration (**Fig. 2e**). Beyond 5 days of administration, continued Dox treatment did not significantly enhance the induction of MHC positive myocytes (**Fig. 2e**), revealing that 5–6 days of Dox administration was sufficient to commit MyoD-hiPSCs to a myogenic lineage. Next, we investigated the effect of initiating Dox administration on various days. Although Dox administration on d0 resulted in a large amount of cell deaths, early Dox administration resulted in highly efficient differentiation (**Fig. S3c, d**). Using these optimized parameters, we established a standardized protocol for myogenic differentiation of MyoD-hiPSCs (**Fig. 3a**).

Applying this differentiation protocol, all MyoD-hiPSC clones derived from 3 distinct parental hiPSC lines could be promoted to form MHC positive myotubes (**Fig. 3b**). The efficiency was calculated from the percentage of MHC positive cells among total cells, and consistently ranged from 70% to 90%, irrespective of the clone of origin (**Fig. 3c**). Differentiated MyoD-hiPSCs changed their shape to spindle-like, uniformly (**Movie S1**). Thus, dominant drug-regulated expression of exogenous *MYOD1,* combined with our differentiation culture conditions overcomes clonal variation, and actualizes efficient and uniform myogenic differentiation.

### Differentiation of Myocytes from MyoD-hiPSCs Occurs Directly

**Figure 4:**
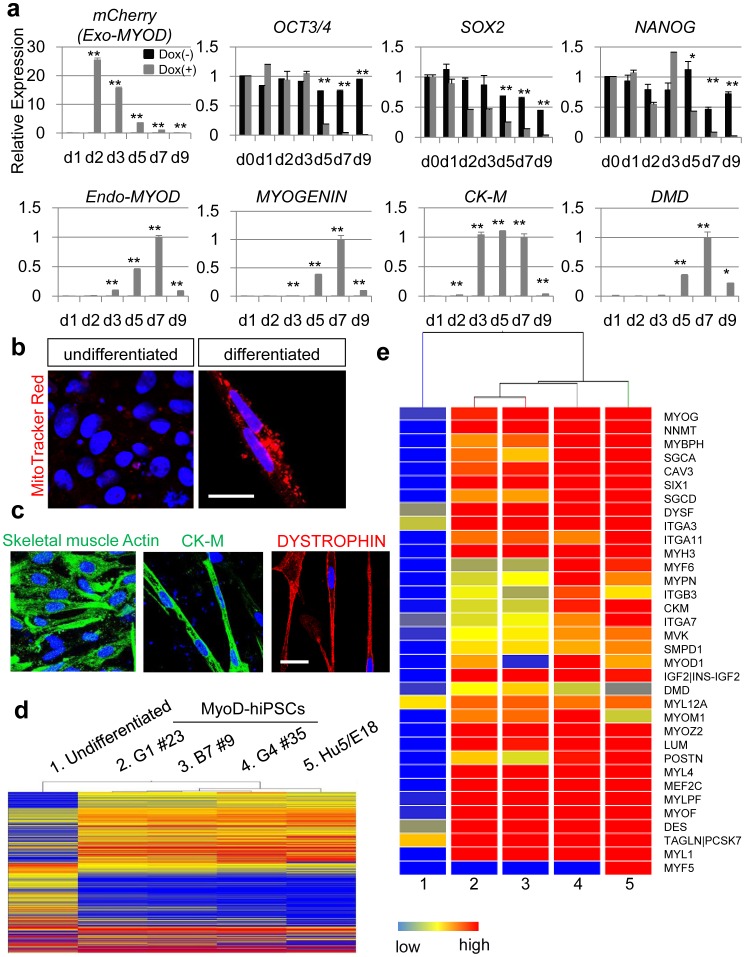
Characterization of myofiber derived from MyoD-hiPSCs. (**a**) Time course gene expression profile for undifferentiated and myogenic markers in B7 #9 MyoD-hiPSC clone with (gray bars) or without (black bars) Dox administration (n = 3). Data are listed as mean±S.D. The data were standardized by β-actin using teratoma. The data on d0 = 1 in undifferentiated markers, such as *OCT3/4*, *SOX2* and *NANOG*. The data on d7 = 1 in other analyses. *: *p*<0.05, **: *p*<0.01, respectively, between Dox(–) and Dox(+). (**b**) Intracellular localization of mitochondria in both undifferentiated and differentiated MyoD-hiPSCs. Scale bar = 20 µm. (**c**) Immunohistochemistry of differentiated MyoD-hiPSCs for mature myogenic markers, such as CK-M, creatine kinase muscle isoform, Skeletal muscle Actin, and DYSTROPHIN. Scale bar = 20 µm. (**d**) Heat map of global mRNA expression comparing undifferentiated hiPSC (sample 1) and differentiated myogenic cells (samples 2-5). (**e**) Myogenic gene profile and unsupervised clustering based on markers associated with myofibers for undifferentiated hiPSCs and differentiated myogenic cells. Red color indicates up-regulated genes and blue color indicates down-regulated genes in (**d**) and (**e**).

To characterize cell responses to this updated differentiation protocol, we analyzed the time course of gene expression of both undifferentiated and myogenic markers in the presence or absence of Dox (**Fig. 4a**). Expression of mCherry, synonymous with exogenous *MYOD1* expression, was the highest at d2 and declined throughout the protocol. As expected, the expression of markers of the undifferentiated markers [22], such as *OCT3/4*, *SOX2* and *NANOG*, gradually decreased through the course of differentiation (**Fig. 4a** **, gray bars**). The expression of endogenous *MYOD1* and *MYOGENIN* [23], both of which are directly and positively regulated by MYOD1, appeared at d3 and peaked at d7 (**Fig. 4a** **, gray bars**). Furthermore, mature myofiber markers such as *creatine kinase muscle isoform* (*CK-M*) [24] and *DYSTROPHIN (DMD)* [25], were also detected following exogenous *MYOD1* expression (**Fig. 4a** **, gray bars**). By contrast, prolonged expression of genes characteristic lacking differentiation and an absence of myogenic gene expression were observed in the absence of Dox administration (**Fig. 4a** **, black bars**). Similar to observations in mouse ESCs [15], Dox-induced *MYOD1* expression in hiPSCs is sufficiently potent to directly promote myogenic differentiation, even in completely undifferentiated cells. In development, myogenic cells derive from a progenitor of mesodermal origin. To further assess whether Dox-induced myogenic differentiation proceeded *via* mesodermal differentiation, expression of mesodermal marker genes was analyzed. The pan-mesodermal marker *BRACHYURY* (*T*) [26], paraxial mesodermal markers *MESP2* [27] and *TBX6* [28], and a dermomyotome marker *PAX3* [29] were expressed transiently during Dox-induced differentiation, whereas in the absence of Dox very low expression of mesodermal genes was observed (**Fig. S4**). To determine whether mesodermal gene expression is essential for myogenic differentiation in our system, siRNA was used to suppress *T* or *TBX6* during the early phase of differentiation. Expression of *T* or *TBX6* was strongly suppressed on d3 and d5 by 48 h of pretreatment with siRNA (**Fig. S5a, b**). Furthermore, both *T* and *TBX6* siRNA treatments indirectly suppressed expression of *PAX3*, which is upstream of *MYOD1* [30]. Despite suppression of mesodermal gene expression, efficiencies of myogenic differentiation were not affected (**Fig. S5c, d**). Thus, although several mesodermal genes express transiently, MyoD-hiPSCs successfully differentiate along a myogenic lineage independent of these factors, seemingly circumventing a mesodermal intermediate stage.

### Myocytes Derived from MyoD-hiPSCs Resemble Mature Myocytes *in vivo*

To assess myogenic properties of differentiated MyoD-hiPSCs, histological and gene expression analyses were performed. Although undifferentiated hiPSCs had few mitochondria, differentiated MyoD-hiPSCs had many mitochondria surrounding their nuclei (**Fig. 4b**). Furthermore, differentiated MyoD-hiPSCs expressed the mature myocyte markers, DYSTROPHIN, skeletal muscle Actin and CK-M **(** **Fig. 4c**). Skeletal muscle Actin does not appear in cardiac muscle and is therefore used to distinguish skeletal myocytes from cardiomyocytes. These features suggest that Dox-induced myogenic cells derived from MyoD-hiPSCs have characteristics of mature myocytes. To further define myogenic cell identity, we analyzed global gene expression profiles of differentiated MyoD-hiPSCs, comparing these with the differentiated immortal human myoblast cell line Hu5/E18 [31], [32], and undifferentiated hiPSCs (**Fig. 4d, e**). The microarray profiles of differentiated MyoD-hiPSCs were similar to those of differentiated Hu5/E18 and quite divergent from those of undifferentiated hiPSCs (**Fig. 4d**). Multiscale bootstrap clustering analysis showed that MyoD-hiPSCs derived myocytes had statistically significant different gene expression profiles from those of undifferentiated hiPSCs and similar gene expression profiles to those of Hu5/E18 derived myocytes (**Fig. S6**). Furthermore, we selected specific genes associated with muscle differentiation and analyzed mRNA expression profiles. Differentiated MyoD-hiPSCs showed high expression levels of the selected muscle associated genes, similar to differentiated Hu5/E18 cells (**Fig. 4e**), with the exception of *MYF5* [33], an upstream transcription factor regulating *MYOD1* (**Fig. 4e**). These data were confirmed by quantitative real time PCR. Consistent with the results of microarray analysis, myogenic transcription factors such as *MYOD1*, *MYOGENIN*, *MEF2C* [34] and *SIX1* [35], which are downstream genes of *MYOD1*, were upregulated in induced cells but not in undifferentiated cells. By contrast, *MYF5*, which is an upstream gene of *MYOD1*, was not upregulated in induced cells (**Fig. S7**). Taken together, Dox-induced myogenic cells generated from MyoD-hiPSCs appear to represent mature myocytes similar to differentiated human myoblasts. Yet, unlike human myoblasts, MyoD-hiPSCs do not express *MYF5*, and may transit directly from undifferentiated into *MYOD1* positive myogenic cells.

### Functional Properties of Dox-induced Myocytes Derived from MyoD-hiPSC

**Figure 5:**
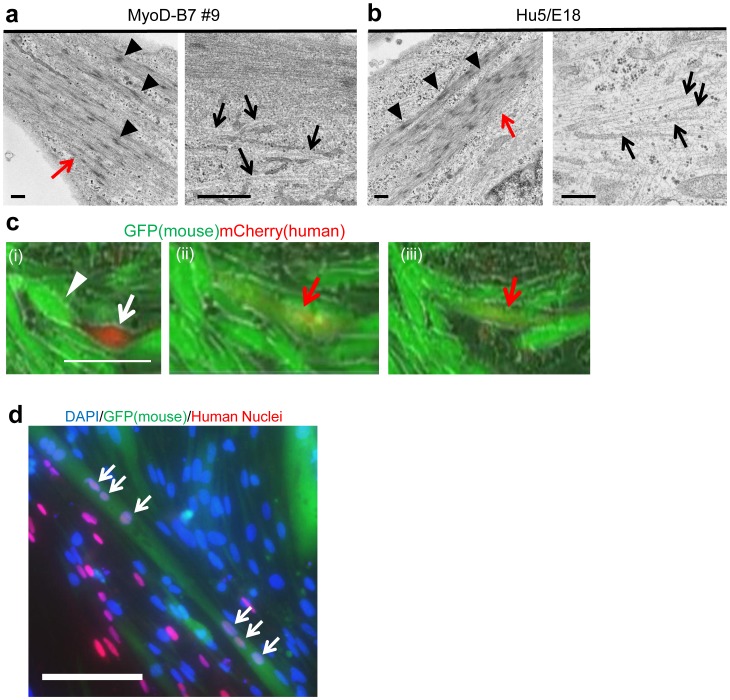
Functional assay for differentiated MyoD-hiPSCs. (**a, b**) Electron microscopy of differentiated MyoD-hiPSCs (**a**) and differentiated human myoblast Hu5/E18 cells (**b**). Red arrows indicate myofibrils. Black arrowheads indicate future Z lines. Black arrows indicate myosin fibers. Scale bars = 500 nm. (**c**) Serial photographs of differentiated MyoD-hiPSCs co-cultured with C2C12 cells. A hiPSC-derived mCherry+ cell (white arrow) fused with a mouse-derived GFP+ cell (white arrowhead) resulting in a yellow cell (red arrow). Time increments between images = TIME. Scale bar = 100 µm. (**d**) Immunohistochemistry of MyoD-hiPSCs co-cultured with C2C12 cells. White arrows indicate human nuclei in a GFP+ murine myofiber. Scale bar = 100 µm.

Structural analysis by electron microscopy revealed that differentiated MyoD-hiPSCs have myofibrils containing future Z line-like structures, and myosin fibers similar to differentiated Hu5/E18 (compare **Fig. 5a, b**). To assess whether such structural properties are sufficient to mediate contraction, Dox-induced myogenic cells were electrically stimulated. As predicted, *MYOD1* induced myotubes could contract coincident with electrical pulses (**Movie S2**). Another discriminating characteristic of myogenic cells is cell fusion, leading to multi-nucleated myotubes. With regard to fusion potential, differentiated MyoD-hiPSCs were co-cultured with the mouse myoblast cell line C2C12 [21], engineered to express GFP. MyoD-hiPSCs were induced towards myogenic differentiation for 7 days. On d7, C2C12 cells were seeded onto induced MyoD-hiPSCs. Two days after co-culture, mCherry positive human myogenic cells fused with GFP positive murine myogenic cells were identified by time lapse photography (**Fig. 5c** and **Movie S3**). Several days later, immunohistochemistry revealed that a number of human nuclei in murine myotubes, confirming cell fusion *in vitro* (**Fig. 5d**). Finally, we transplanted differentiated MyoD-hiPSCs into tibialis anterior muscles (TA muscle) of non-obese diabetic/severe-combined immunodeficient-duchenne muscular dystrophy null (NOD/scid-DMD) mice [36] (**Methods**). On d28 after transplantation, although the number of signals was a few, specific staining with anti-human spectrin (**Fig. S8a**) and anti-human dystrophin (**Fig. S8b**) was detected in mouse TA muscles. These results indicate that MyoD-hiPSC-derived myocytes display fusion potential both *in vitro* and *in vivo*. Taken together, Dox-induced myogenic cells derived from MyoD-hiPSCs achieve the functional properties of muscle, similar to differentiated human myoblasts.

### Application of Myogenic Differentiation to Disease Modeling

**Figure 6:**
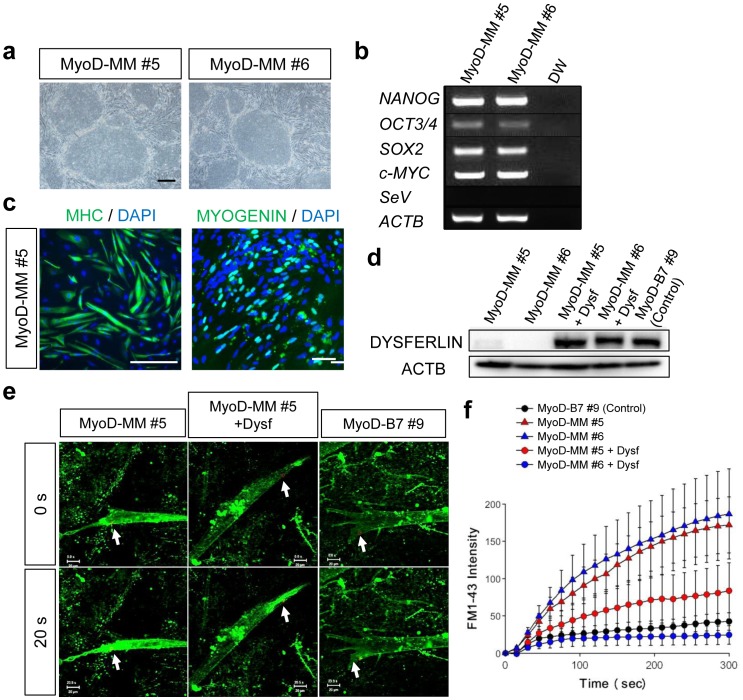
Modeling Miyoshi Myopathy (MM) by patient derived-hiPSCs. (**a**) Morphology of patient derived MM-hiPSC clones, expanded following G418 selection for Tet-MyoD1 vector transposition. Scale bar = 200 µm. (**b**) RT-PCR analysis of endogenous pluripotent stem cell markers in MyoD-MM hiPSCs. (**c**) Efficient myogenic differentiation of MyoD-MM hiPSCs according to the protocol defined in Figure 3a. MHC positive (left), or Myogenin positive (right) cells were observed dominantly. Scale bars = 100 µm. (**d**) DYSFERLIN expression of the myofibers from MyoD-MM hiPSCs (lane 1, 2), rescued MyoD-MM hiPSCs which expressed full-length *DYSF* cDNA driven by EF1α promoter (lane 3, 4), and control non-diseased MyoD-hiPSCs (lane 5) confirmed by western blotting. ACTB = β-actin. (**e**) Entry of FM1-43 green fluorescent dye into differentiated myofibers from MyoD-MM #5 (left), rescued MyoD-MM #5 with *DYSF* expression (middle), or control MyoD-hiPSC clone B7 #9 (right), before (0 s) and 20 s after (20 s) two photon laser-induced damage of the sarcolemmal membrane (arrow). Scale bars = 20 µm. (**f**) Summary time course data of accumulation of FM1-43 dye in laser-damaged myofibers derived from B7 #9 (black circles), MyoD-MM hiPSCs (red or blue triangles) and rescued MyoD-hiPSCs with DYSFERLIN expression (red or blue circles). n = 5 for each clone. Data are listed as mean ±S.E.

We applied our myogenic induction system to assess the utility of these differentiated cells in modeling the human disease Miyoshi myopathy (MM) [16]. MM hiPSCs were generated from MM patient fibroblasts by transduction of the 4 Yamanaka factors (*OCT4*, *SOX2*, *KLF4* and c-*MYC*) with Sendai virus (SeV) vectors (**Methods**). Subsequently, we introduced the Tet-MyoD1 vector into MM hiPSCs, and chose 2 independent clones (MyoD-MM #5 and #6) for further analysis. These 2 clones were morphologically identical to the other hiPSCs (**Fig. 6a**), expressed endogenous pluripotency marker genes without detectable persistence of the SeV viral RNA genome (**Fig. 6b**), and formed teratomas *in vivo* (**Fig. S9**). MyoD-MM hiPSCs were unhindered in their ability to differentiate into MHC or MYOGENIN positive mature myocytes (**Fig. 6c**). Yet, differentiated MyoD-MM cells demonstrated impaired expression of DYSFERLIN [17], [18], as expected from the primary genetic lesion (**Fig. 6d, lanes 1, 2**). Thus, we derived rescued MyoD-MM+Dysf hiPSC clones by stable transgenic over-expression of DYSFERLIN, and confirmed DYSFERLIN expression in 2 rescue clones and the MyoD-B7 #9 control line by western blotting (**Fig. 6d**).

To recreate pathological conditions, we assessed membrane repair function in both diseased and DYSFERLIN-rescued MyoD-MM hiPSCs compared to control MyoD-hiPSCs (**Fig. 6e, f**). A myotube from MyoD-MM clone #5 displayed extensive uptake of FM1-43 in all cytoplasmic lesions, indicating defective membrane repair following two-photon laser-induced injury of the sarcolemma [17] (**Fig. 6e left**, **Movie S4**). In contrast, myotubes from MyoD-MM #5+ Dysf and MyoD-B7 #9 control cells display focal uptake of FM1-43 at the damaged area (**Fig. 6e** **center and right panels, Movie S5 and S6**). Indeed, the apparently unimpeded uptake of FM1-43 observed in MM patient-derived myotubes, was reversed by over-expression of *DYSF,* suggesting efficient membrane resealing, similar to control cells (**Fig. 6f**). Thus, in vitro differentiated MM patient iPSCs faithfully recapitulated the expected pathological condition, which could be reversed by overexpression of the affected gene product, suggesting a possible treatment for MM by using gene therapy.

## Discussion

Over the years, engineered model mice have been the mainstay in understanding disease pathology [36], [37], [38]. Yet, due to discrepancies between mouse phenotypes and actual human disease pathology [39], it would be preferable to evaluate disease and perform *in vitro* screening for potential therapeutics in a reliable human system. In considering *in vitro* disease modeling and screening, large numbers of cells are necessary to generate and reproduce experimental data. Fibroblasts and many other somatic cells are not able to proliferate indefinitely and quickly reach senescence, failing to maintain a similar level of quality throughout their limited replicative lifespan. As hiPSCs have a clear advantage as an indefinitely self-renewing cell source for *in vitro* assays, iPSC technology coupled with robust *in vitro* differentiation has been hailed as a major breakthrough in the field [40]. Myogenic induction from hiPSCs, following the protocols outlined here, provides consistent quality, exhibiting higher efficiency and reproducibility than described in previous reports [10], [12], whereas the period required for induction is as short as 10 days. These are significant improvements and represent major steps towards iPSC-based disease-modeling and drug screening for myogenic disorders.

Although forced expression of *MYOD1* by mRNA treatment can induce myocytes from hiPSCs-derived fibroblasts, the efficiency of myogenic differentiation is limited to less than 40% [12]. This may in part be due to a low efficiency of mRNA transfection in fibroblasts. In contrast, the *piggyBac* vector system employed here presents advantages in obtaining transgenic hiPSCs through efficient and stable genomic integration, a suitable approach for *in vitro* applications. Transgenic hiPSC populations or clones carrying the *piggyBac* vector may be easily purified before Dox induction by using simple drug selection. Clones derived in this manner display robust and uniform drug-induced transgene activation through the early stages of differentiation, ensuring an effective and coordinated response, and thus achieving efficiencies in excess of 70%. Furthermore, the *piggyBac* Tet-inducible gene expressing vector is almost completely inactive in partially or fully differentiated hiPSCs, but expresses strongly in undifferentiated hiPSCs. By initiating differentiation using the master regulator, MYOD1, in homogenous undifferentiated hiPSCs, rather than a heterogenous differentiated cell population, our approach achieves highly consistent efficiency. Recently, several reports have demonstrated highly efficient myogenic differentiation of hiPSCs driven by the overexpression of transcription factors [6], [13], [14]. Darabi et al. showed that PAX7-overexpression in mesodermal cells differentiated from hiPSCs through embryoid body formation, could promote efficient myogenic differentiation and generate transplantable myogenic progenitors which were applicable for muscle regeneration. However, their method of differentiation entailed many complicated steps including cell sorting by flow cytometer, and the time taken to induce mature myocytes was more than 4 weeks [6]. Tedesco et al. demonstrated that human iPSC-derived mesoangioblast-like progenitors (HIDEMs) could be generated by a 3 weeks, multi-step differentiation method. Although, the HIDEMs were able to act as transplantable myogenic progenitors, with excellent regeneration potentials in impaired muscle, they had poor myogenic differentiation potential *in vitro*; efficient *in vitro* myogenic induction of the HIDEMs depended on forced expression of *MYOD1* from a tamoxifen-inducible lenti-viral vector system [13]. Goudenege et al. demonstrated that efficient myogenic differentiation could be induced from hiPSC-derived mesenchymal cells by adeno-viral transduction of *MYOD1* [14]. The use of an integration-free adenoviral system could be advantageous for muscular disease cell therapy, but it could potentially be a complicated step during establishment of drug screening platforms following disease modeling. In all 3 previously reported procedures, hiPSCs were differentiated into mesodermal or mesenchymal progenitors prior to forced expression of the transcription factors. Although, these procedures able to induce myogenic progenitors for use in cell therapy, pre-differentiation steps, with serum-containing medium, could increase the clonal variation of hiPSCs, and consequently affect the reproducibility of the methods. By contrast, we initiated differentiation in undifferentiated hiPSCs, which are relatively homogenous, and our simple method exhibits high reproducibility. Therefore, our method has advantages for application in drug screening following muscular disease modeling by using patient-derived hiPSCs, as a simple, rapid, and reproducible myogenic differentiation protocol, compared to previously reported methods.

Recently, it was revealed that premyogenic mesodermal genes are activated by transgenic expression of *MYOD1* in undifferentiated mouse embryonal carcinoma cells [11]. In our hiPSC differentiation method, exogenous *MYOD1* acts as a dominant regulator of myogenesis, through activation of recognized mature myogenic gene networks, presumably without requiring stepwise mesodermal differentiation as occurs during embryonic development. We confirm that exogenous expression of *MYOD1* in undifferentiated hiPSCs can indeed promote premyogenic mesodermal gene expression. However, our knockdown experiments suggest that premyogenic mesodermal gene expression is dispensable, and that the majority of the undifferentiated hiPSC population can be directly differentiated into mature myocytes. This observation supports our hypothesis that Dox-induced *MYOD1* expression in hiPSCs directly activates the myogenic gene network independent of premyogenic mesodermal genes.

Myocytes induced from hiPSCs with *MYOD1* demonstrate similar global gene expression patterns and microstructures to the immortal human myoblast cell line Hu5/E18. Moreover, induced myocytes acquire mature functional properties such as cell fusion and twitching on electrical stimulation. This report is the first to present such detailed analysis for the functional properties of induced myocytes, which will be absolutely critical in attaining reliable muscular disease modeling. We applied our system to rproduce pathological conditions of MM by using patient-derived hiPSCs. We observed defective membrane repair in MM hiPSC-derived myotubes after two-photon laser-induced injury of the sarcolemma, and rescued this phenotype by transgenic expression of full-length *DYSF*. It is still unclear why defective membrane repair may result in chronic muscle inflammation in MM patients. Importantly, DYSFERLIN is also expressed in immune cells [41], and there is some debate as to which cell lineage (myocytes or immune cells) is responsible for the pathology of MM. As immune cells may be differentiated directly from iPSCs [42], this question may be addressed by co-culture of healthy iPSC-derived immune cells and MM patient iPSC-derived myocytes, or vice versa. In this context, our differentiation system should prove useful in deriving enriched myocyte populations for co-culture, and help to resolve the remaining questions regarding MM pathology.

Myogenic differentiation of hiPSCs provides clear advantages over the use of disease myocytes or fibroblasts in recreating pathological conditions, and the *in vitro* study of myogenic disorders. Our defined differentiation system provides reproducibility, high efficiency and short induction periods. These properties could help promote the establishment of human muscular disease models, which would eventually lead to a better understanding of disease development. Furthermore, drug-screening platforms based on human cells will help resolve incongruent drug treatment efficacies observed between mice and human, hopefully leading to prospective treatments and eventually cures.

## Methods

### Cell Culture

Human iPSC lines 201B7, 253G1 and 253G4 were kindly provided by Dr. Shinya Yamanaka [1]. Human dermal fibroblasts used for generating 201B7 and 253G4 were purchased from Cell Applications, Inc. All human iPS cells were established according to procedures approved by the Ethics Committee on Human Stem Cell Research of the Institute for Frontier Medical Sciences, Kyoto University. The ethics committee approved our studies. The hiPSCs were cultured in human iPS medium composed of primate ES medium (ReproCELL) supplemented with 4 ng/mL recombinant human basic fibroblast growth factor (bFGF, Wako), and otherwise maintained as previously described [1]. The immortalized human myoblast cell line Hu5/E18 provided by RIKEN BRC through the National Bio-Resource Project of the MEXT, Japan, was maintained and differentiated as described previously [32].

### Generation of an iPS Cell Line Derived from a Patient with Miyoshi Myopathy (MM)

Studies were approved by the authors’ Institutional Review Board and conducted under the Declaration of Helsinki. MM patient information was encoded to protect privacy, and written informed consent obtained. The MM patient was known to have 2 mutations in the *DYSF* gene. Fibroblasts from the patient were cultured from skin biopsy explants under protocols approved by the Ethics Committee on Human Stem Cell Research of the Graduate School of Medical Sciences, Kumamoto University. The ethics committee specifically approved these studies. The patient provided written informed consent to participate in these studies. Skin samples were minced and cultured in Dulbecco’s Modified Eagle’s Medium (DMEM; Invitrogen) supplemented with 10% fetal bovine serum (Invitrogen). Patient-derived hiPSCs were generated with SeV as described previously [43] and are referred to herein as MM-hiPSCs.

### Generation of a Doxycycline-inducible MyoD-hiPS Cell Line

A cDNA clone of *MYOD1* was purchased from MGC clone (Invitrogen, MGC:71135, GenBank: BC064493.1). The coding region of *MYOD1* was PCR cloned into pENTR Directional TOPO (Invitrogen) according to the manufacturer’s protocol. The PB-TAC-ERN (KW111) vector was assembled with standard cloning methods by using PB-TET [19] as a backbone. The βgeo reporter in PB-TET was swapped for mCherry, and a constitutive rtTA-neo expression cassette inserted. pVITRO1-neo (InvivoGen) was introduced at the 3′ end of the construct. Details of the cloning steps are available upon request. Using Gateway cloning, the *MYOD1* cDNA was transferred to the PB-TAC-ERN destination vector as indicated in **Fig. 1a**, to yield the transposon PB-MyoD1.

Plasmid DNA for transfection was prepared using a QIAprep Spin Miniprep Kit (Qiagen). Non-diseased control hiPSCs and MM-hiPSCs were seeded onto mitomycin C-treated SNL feeder cells [1] in a 6-well culture dish. The next day, 1 µg destination vector and 1 µg PBase plasmid [19] were transfected into hiPSCs with FuGENE HD (Roche), according to the manufacturer’s protocol. Forty-eight hours after transfection, 100 µg/mL G418 (Nacalai Tesque) was added to select for stable Tet-MyoD1 transposition, clones were picked, and the appropriate MyoD-hiPSC clones with high mCherry expression selected.

### Overexpression of *DYSFERLIN* in MM Patient-derived iPSCs

Full-length *DYSF* cDNA was kindly provided by the Jain Foundation. The *DYSF* cDNA was Gateway cloned into a PB-based, EF1α promoter-driven constitutive expression vector which co-expresses puromycin resistance (PB-Dysferlin). MyoD-MM-hiPSCs were seeded onto mitomycinC-treated SNL-PH feeder cells (resistant to neomycin, puromycin, and hygromycin). The next day, 1 µg of both PB-Dysferlin and PBase [19] plasmids were transfected into MyoD-MM-hiPSCs by using FuGENE HD (Roche), as described above. Forty-eight hours after transfection, 100 µg/mL G418 (Nacalai Tesque) and 1 µg/mL Puromycin (Nacalai Tesque) were added to select for cells carrying both the Tet-MyoD and PB-Dysferlin vectors. After selection, MyoD-MM+Dysferlin hiPSC clones displaying DYSFERLIN expression were selected by western Blotting.

### Differentiation of MyoD-hiPSCs

MyoD-hiPSCs were seeded onto CollagenI (Iwaki) or Matrigel (BD Biosciences) coated dishes without feeder cells. Matrigel was diluted 1∶50 with primate ES medium. MyoD-hiPSCs were trypsinized and dissociated into single cells. The cell number plated ranged from 2.0×10^5^ to 1.0×10^6^ per 10cm^2^. Culture medium was changed to human iPS medium without bFGF and with 10 µM Y-27632 (Nacalai Tesque). After 24 h, 1 µg/mL doxycycline (LKT Laboratories) was added to the culture medium. After an additional 24 h, culture medium was changed to differentiation medium composed of alpha Minimal Essential Medium (αMEM; Nacalai Tesque) with 5% KSR (Invitrogen), 50 mU/L Penicillin/50 µg/L Streptomycin (Invitrogen), and 100 µM 2-Mercaptoethanol (2-ME). After an additional 5 days, culture medium was changed to DMEM with 5% horse serum (Sigma), 50 mU/L Penicillin/50 µg/L Streptomycin, 10 ng/mL recombinant human insulin-like growth factor 1 (Peprotech), 2 mM L-Glutamine and 100 µM 2-ME. Approximately 2 days later, myogenic properties were assessed.

### Fluorescence Activated Cell Sorting Analysis of mCherry Positive Cells

Doxycycline-treated cells were washed in Phosphate buffered saline (PBS) and incubated for 5 min with 0.25% Trypsin to dissociate into single cells. After cells were counted, they were suspended in Hank’s balanced salt solution (Life technologies), supplemented with 1% BSA at 1.0×10^6^ cells/100 µL and analyzed on an LSR Fortessa (BD Biosciences) for the expression of mCherry.

### RNA Isolation and Reverse Transcription

Total RNA was isolated using Sepazol (Nacalai Tesque) according to the manufacturer’s protocol. Residual genomic DNA was digested and removed using DNase I (Invitrogen) treatment. First strand cDNA was synthesized using the Superscript III First-Strand Synthesis System (Invitrogen) and random hexamer primers or oligo (dT) according to experimental aims. Reverse transcription and conventional PCR were performed as described previously [1]. Quantitative PCR was performed using probe sets, SYBR Green (Applied Biosystems), and Step One thermal cycler (Applied Biosystems). β-actin was used as an internal controls. For standardization, we set the value of the d0, d5 or d7 sample as the control value ( = 1.0). Primers used in this study are listed in **Table S1**.

### Transplantation Studies

All mouse experiments were carried out according to protocols approved by the Animal Research Committee of Kyoto University. The committee specifically approved these animal studies. NOD/Scid mice were purchased from Charles River Laboratories, and were mated with DMD-null mice (which do not express DYSTROPHIN) to generate the NOD/Scid-DMD mice used for *in vivo* transplantation studies. Mice were anesthetized with diethyl ether and injured with cardiotoxin before intramuscular cell transplantation. Twenty-four hours after cardiotoxin damage, d6 Dox-treated MyoD-hiPSCs (1.0×10^6^–9.5×10^6^ cells per 50 mL 10% Matrigel in αMEM) were injected into left TA muscles. All mice used in this study were humanely sacrificed 28 days after transplantation and tissue samples were collected. Collected samples were embedded into a pedestal of tragacanth gum.

Engrafted muscles were frozen in isopentane cooled in liquid nitrogen. Serial cryosections (10–20 µm) were collected. Tissue cryosections were fixed and stained as described previously [44]. Briefly, tissue cryosections were fixed using 4% paraformaldehyde (PFA)/PBS for 20 min. Samples were blocked with 1% Goat serum (Sigma), 0.1% Bovine serum Albumin (Sigma), 0.2% Triton X-100 (Nacalai Tesque)/PBS for 60 min. Samples were incubated with primary antibodies for 16–18 h at 4°C. Next day, samples were washed 3 times in PBS and incubated with secondary antibodies for 1 h at room temperature. 4,6-Diamidino-2-phenylindole (DAPI; 1∶5000) was used to counter-stain nuclei. PermaFluor (Thermo Scientific) was used as a mounting agent. Samples were observed by LMS710 confocal microscopy (Carl Zeiss).

### Teratoma Formation Assay

For teratoma formation, sub-confluent undifferentiated human iPSCs were harvested and resuspended in maintenance medium containing 50% Matrigel. Human iPSCs were injected into tibialis anterior muscles of NOD/scid mice by using pre-chilled syringe with a 27G needle. Mice were sacrificed for assays 4 weeks after transplantation.

### Co-culture with C2C12 Cells

Differentiated MyoD-hiPSCs were co-cultured with a C2C12 cell line which expresses GFP constitutively from the CAG promoter (kindly provided Dr. Kurisaki, Department of Growth Regulation, Institute for Frontier Medical Sciences, Kyoto University). MyoD-hiPSCs were differentiated for 7 days according our protocol. On d7, medium was replaced with DMEM supplemented with 5% horse serum, and 1.0×10^5^ C2C12 cells were seeded onto MyoD-hiPSCs. Bio Station CT (Nikon) was used for time-lapse observation of co-cultured samples. The duration of image acquisition was 1 h.

### Immunohistochemistry of Cultured Cells

Cells were fixed and stained as described previously [1]. Briefly, cells were fixed using 2% PFA/PBS. Samples were blocked with 5% Blocking One (Nacalai Tesque)/PBS for 30 min and then incubated with primary antibodies diluted in 5% Blocking One/PBS for 16–18 h at 4°C. Next, samples were washed 3 times in PBS and incubated with secondary antibodies diluted in 5% Blocking One/PBS for 1 h at room temperature. DAPI (1∶5000;Sigma) was used to counter-stain nuclei. Antibodies are listed below. For mitochondrial staining, MitoTracker Red CMXRos (Invitrogen) was used according to the manufacturers protocol. Samples were observed with a BZ-9000E (Keyence).

### Western Blotting

Cells were harvested and analyzed as described previously [1]. Briefly, semiconfluent cells were lysed in RIPA buffer (50 mM Tris-HCl, pH 8.0, 150 mM NaCl, 1% Nonidet P-40 (NP-40), 1% sodium deoxycholate, and 0.1% SDS), supplemented with protease inhibitor cocktail (Roche). Cell lysates (10 µg) were separated by electrophoresis on 8% or 12% SDS-polyacrylamide gel and transferred to a polyvinylidine difluoride membrane (Millipore). The blot was blocked with TBST (20 mM Tris-HCl, pH 7.6, 136 mM NaCl, and 0.1% Tween-20) containing 1% skim milk and then incubated with primary antibody solution at 4°C overnight. After washing with TBST, the membrane was incubated with secondary antibody for 1 h at room temperature. Signals were detected with Immobilon Western chemiluminescent HRP substrate (Millipore) and ChemiDoc XRS+imaging system (BIO-RAD). Antibodies are listed below.

### Primary and Secondary Antibodies

Primary antibodies used in this study were as follows: rat, anti-Laminin monoclonal antibody (mAb; 1∶15; Alexis), mouse Anti-human Myosin heavy chain mAb (MF20; 1∶400; R&D), mouse anti-myogenin mAb (F5D; 1∶400; Santa Cruz), rabbit anti-MyoD polyclonal antibody (pAb; M-318, 1∶400; Santa Cruz), mouse anti-human Spectrin mAb (1∶100; Leica), mouse anti-human nuclei mAb (1∶200; Millipore), mouse anti-alpha skeletal muscle actin mAb (1∶200; Acris), rabbit anti-Creatine Kinase M pAb (Y14; 1∶100; Bioworld Technology), mouse anti-Dysferlin mAb (NCL-Hamlet, 1∶25; Leica), mouse anti-Dystrophin mAb (NCL DYS1, 1∶100; Leica), mouse anti-SSEA-4 mAb (1∶100; Millipore), mouse anti-TRA-1-81 mAb (1∶100; Millipore), and mouse anti-Nanog mAb (1E6C4, 1∶2000; Cell Signaling).

Secondary antibodies used in this study were as follows: Alexa Fluor 488 conjugated goat-anti-rabbit or mouse IgG, Alexa Fluor 568 conjugated goat-anti- rabbit or mouse IgG (1∶500; Invitrogen), and HRP conjugated goat-anti-mouse IgG (1∶200 for IHC, 1∶2000 for western blotting; Vector).

### Statistical Analysis

Differences between samples were assessed by using the Student’s two-tailed *t* test for independent samples.

### Electron Microscopy

Samples were chemically fixed and observed by Tokai Electron Microscopy, Inc. according to their protocol (Tokai Electron Microscopy, Inc.). Briefly, Samples were fixed with 2% PFA/PBS, 2% glutaraldehyde (GA) in 0.1 M phosphate buffer (Pb), pH 7.4 at 37°C and put them into a refrigerator (4°C) for 30 min. Thereafter, they were fixed with 2% GA in 0.1 M Pb at 4°C overnight. After these fixation the samples were rinsed 3 times with 0.1 M Pb for 30 min each, followed by post fixation with 2% osmium tetroxide (OsO4) in 0.1 M Pb at 4°C for 1 h.

The samples were dehydrated through a series of graded ethanol (50%, 70%, 90%, and 100%). The schedule was as follows: 50% and 70% for 5 min each at 4°C, 90% for 5 min at room temperature, and 3 changes of 100% for 5 min each at room temperature.

The samples were transferred to a resin (Quetol-812; Nisshin EM Co., Tokyo, Japan), and polymerized at 60°C for 48 h.

The blocks were ultra-thin sectioned at 70 nm with a diamond knife using a ultramicrotome (ULTRACUT UCT; Leica) and sections were placed on copper grids. They were stained with 2% uranyl acetate at room temperature for 15 min, and then rinsed with distilled water followed by being secondary-stained with Lead stain solution (Sigma-Aldrich Co.,) at room temperature for 3 min.

The grids were observed by a transmission electron microscope (JEM-1200EX; JEOL Ltd.,) at an acceleration voltage of 80 kV. Digital images (2048×2048 pixels) were taken with a CCD camera (VELETA; Olympus Soft Imaging Solutions GmbH).

### Microarray

Total RNA from each condition was obtained from samples by using the RNeasy Mini Kit (Qiagen) according to the manufacturer’s instructions. The quality of RNA samples were determined to be sufficient for Microarray analysis by using the Agilent RNA 6000 Nano Assay (Agilent Technologies). After confirming the quality of RNA, cRNA was synthesized on day one. On d2, cDNA was synthesized and purified. *In vitro* transcription and biotin labeling, and hybridization to the GeneChip ST Array (Affymetrix) were performed according to Affymetrix protocols on d3 and 4. Samples were loaded onto a Fluidics station (Affymetrix) and their signals were analyzed.

### Microarray Data Analysis

Affymetrix CEL files were imported into GeneSpringGX 11.5.1. Probe intensities were normalized, and expression signals of all genes (probe sets) were calculated using RMA 16 as implemented in GeneSpring software. The data were grouped into those of undifferentiated hiPSCs, MyoD-hiPSCs derived myocytes, and Hu5/E18 derived myocytes. Upregulated genes were identified by at least 2-fold changes. A heat map was created using a clustering function algorithm of on both entities and conditions. Distance metric was Euclidean. Linkage rule was single. Myogenic upregulated genes were assembled arbitrarily. As statistical analysis, multiscale bootstrap clustering was performed.

### siRNA Transfection

The siRNAs targeting T, TBX6 and a scrambled negative control were purchased (Sigma, SASI_Hs01_00221962, SASI_Hs01_00166068 and SIC-001-10). siRNA (50 nM) was transfected into hiPSCs seeded at a density of 2.0 to 3.0×10^5^ cells per 10cm^2^ on d0 and d3, respectively, using Lipofectamine RNAiMAX (Invitrogen) according to the manufacturer’s protocol.

### Membrane Repair Assay

Membrane repair assays were performed using differentiated myotubes as described previously [17]. Briefly, membrane damage was induced in the presence of FM1-43 dye (2.5 mM; MolecularProbes) with a two-photon confocal laser-scanning microscope (LSM710NLO; Zeiss) coupled to a 10-W Argon/Ti:sapphire laser. Images were captured beginning 20 s before (t = 0) and for 3 min after irradiation at 10 s intervals. For every image taken, the fluorescence intensity at the site of damage was measured with Zeiss LSM 710NLO imaging software.

### Electrical Stimulation

Electrical stimulation was performed using differentiated MyoD-hiPSCs at around d14 by using C-Dish (ION Optics) as previously described [45]. We loaded electrical voltage with the electric stimulator (Uchida Denshi) at 100 V onto differentiated myotubes for 3 msec at repeated 1 s intervals.

## Supporting Information

Figure S1 **Evaluation of pluripotency of MyoD-hiPSCs.** (**a**) Immunohistochemistry of undifferentiated markers. Scale bar = 100 µm. (**b**) RT-PCR analysis for undifferentiated markers. (**c**) Teratoma formation assay from MyoD-hiPSCs and empty vector transduced hiPSCs. H&E staining of teratoma formed in TA muscle from NOD/scid mouse. Three germ layers formed in teratoma were shown in each panel, respectively. Arrows indicate each germ layer, respectively. Scale bars = 100 µm.
(TIF)

Figure S2 **Evaluation for MyoD-hiPSC clones.** (**a**) Expression of mCherry which is synonymous with exogenous *MyoD1* driven by Dox treatment for 24 h. (**b**) RT-PCR analyses of MyoD-hiPSC clones. Cloned MyoD-hiPSCs had no leaky expression of exogenous *MyoD1* without Dox, while they could express exogenous *MyoD1* 24 h after Dox addition. Endogenous *MYOD1* could be promoted 96 h after Dox addition.
(TIF)

Figure S3 **Other myogenic induction methods by SB-OGs system or changing Dox-addition days.** (**a**) Protocol of myogenic induction via EB outgrowth. (**b**) Expression of mCherry and immunohistochemistry of MHC. Scale bars = 100 µm. (**c**) Protocol of changing the timing of dox-addition. (**d**) The percentage of MHC positive cells per total cells. **p<0.01.
(TIF)

Figure S4 **Expression of premyogenic mesodermal markers.** Quantitative real time PCR for premyogenic markers was performed during MyoD-hiPSC differentiation in B7 #9 MyoD-hiPSC clone with (gray bars) or without (black bars) Dox administration (n = 3). Data are shown as the mean ± SD. The data were standardized by β-actin using embryoid body. The data on d5 or d7 = 1. *p<0.05, **p<0.01, respectively.
(TIF)

Figure S5 **Suppression of mesodermal markers by siRNA.** The siRNA reagent targeting T or TBX6 was added to differentiation culture of MyoD-hiPSCs (clone B7 #9) at d0 or d3 (**a, b**). Quantitative real time PCR was then performed on d3 (**a**) or d5 (**b**) (n = 3). The expression of PAX3 was suppressed by siRNAs for both T and TBX6. *p<0.05 (**c**) The expression of MHC in differentiated MyoD-hiPSCs with or without siRNA treatment on d9. Scale bar = 100 µm. (**d**) Percentage of MHC positive cells 9 days after differentiation with or without siRNA treatment in B7 #9 MyoD-hiPSC clone (n = 3).
(TIF)

Figure S6 **Multiscale bootstrap clustering for the data from microarray.** The number of repeated calculation was 1000 times. Abbreviated words “AU” and “BP” means “apporoximately unbiased p-value” and “bootstrap probability,” respectively. Distance means correlation. Cluster method was average.
(TIF)

Figure S7 **Confirmation of the data obtained from microarray by quantitative real time PCR.** (**a**) Relative gene expression of transcription factors which are significant in microarray analyses. Data are listed as mean ± S.D. The data were standardized by β-actin using teratoma. The data on d0 = 1. The data in *Endo-MYOD1, MYOGENIN, MEF2C* and *SIX1* were expressed with logarithmic Y axes because differentiated cells showed extremely high values, respectively. **p<0.01.
(TIF)

Figure S8 **Fusion potential** ***in vivo.*** Immunohistochemistry of TA muscles from NOD/Scid-DMD mice after 28 days after transplantation of d6 MyoD-hiPSCs. Scale bars = 20 µm. (**a**) Human Spectrin expression (red) was detected along with Laminin (green). (**b**) Human DYSTROPHIN expression (green) was detected along with Laminin (white).
(TIF)

Figure S9 **Teratoma formation assay from MyoD-MM hiPSCs.** (**a**) H&E staining of teratoma formed in TA muscle from NOD/scid mouse. Scale bar = 100 µm. (**b**) H&E staining of three germ layers formed in teratoma. Arrows indicate each germ layer, respectively. Scale bars = 100 µm.
(TIF)

Table S1 **PCR-primers were listed for both RT-PCR and quantitative real-time RT-PCR.**
(DOCX)

Movie S1 **The MyoD-hiPSCs changed their shape to spindle-like uniformly during differentiation from d1 to d7.**
(WMV)

Movie S2 **Contraction of myofiber derived from MyoD-hiPSCs at differentiation d14 by electric stimulation.**
(WMV)

Movie S3 **Fusion of hiPS cells with murine myofiber.** Red shows human and green shows murine derived myogenic cells.
(WMV)

Movie S4 **Membrane repair assay of MyoD-hiPSC derived myofibers from MM patient.** Red circle indicates damaged point.
(WMV)

Movie S5 **Membrane repair assay of MyoD-hiPSC derived myofibers from MM patient with DYSFERLIN over-expression.** Red circle indicates damaged point.
(WMV)

Movie S6 **Membrane repair assay of MyoD-hiPSC derived myofibers from non-disease control.** Red circle indicates damaged point.
(WMV)
