# Correction: Efficient and Reproducible Myogenic Differentiation from Human iPS Cells: Prospects for Modeling Miyoshi Myopathy *In Vitro*

**DOI:** 10.1371/annotation/63972dc9-3a31-43d0-ad52-bc46fd948c03

**Published:** 2013-12-20

**Authors:** Akihito Tanaka, Knut Woltjen, Katsuya Miyake, Akitsu Hotta, Makoto Ikeya, Takuya Yamamoto, Tokiko Nishino, Emi Shoji, Atsuko Sehara-Fujisawa, Yasuko Manabe, Nobuharu Fujii, Kazunori Hanaoka, Takumi Era, Satoshi Yamashita, Ken-ichi Isobe, En Kimura, Hidetoshi Sakurai

An incorrect file for Movie S2 was uploaded with this manuscript. Please view the correct Movie S2 here: http://www.plosone.org/corrections/pone.0061540.001.cn.wmv

