# Supplementary figures and images for: Efficient and Reproducible Myogenic Differentiation from Human iPS Cells: Prospects for Modeling Miyoshi Myopathy In Vitro
Source: PLoS One. 2013 Apr 23;8(4):e61540. doi: 10.1371/journal.pone.0061540 (PMC3633995; doi:10.1371/journal.pone.0061540)

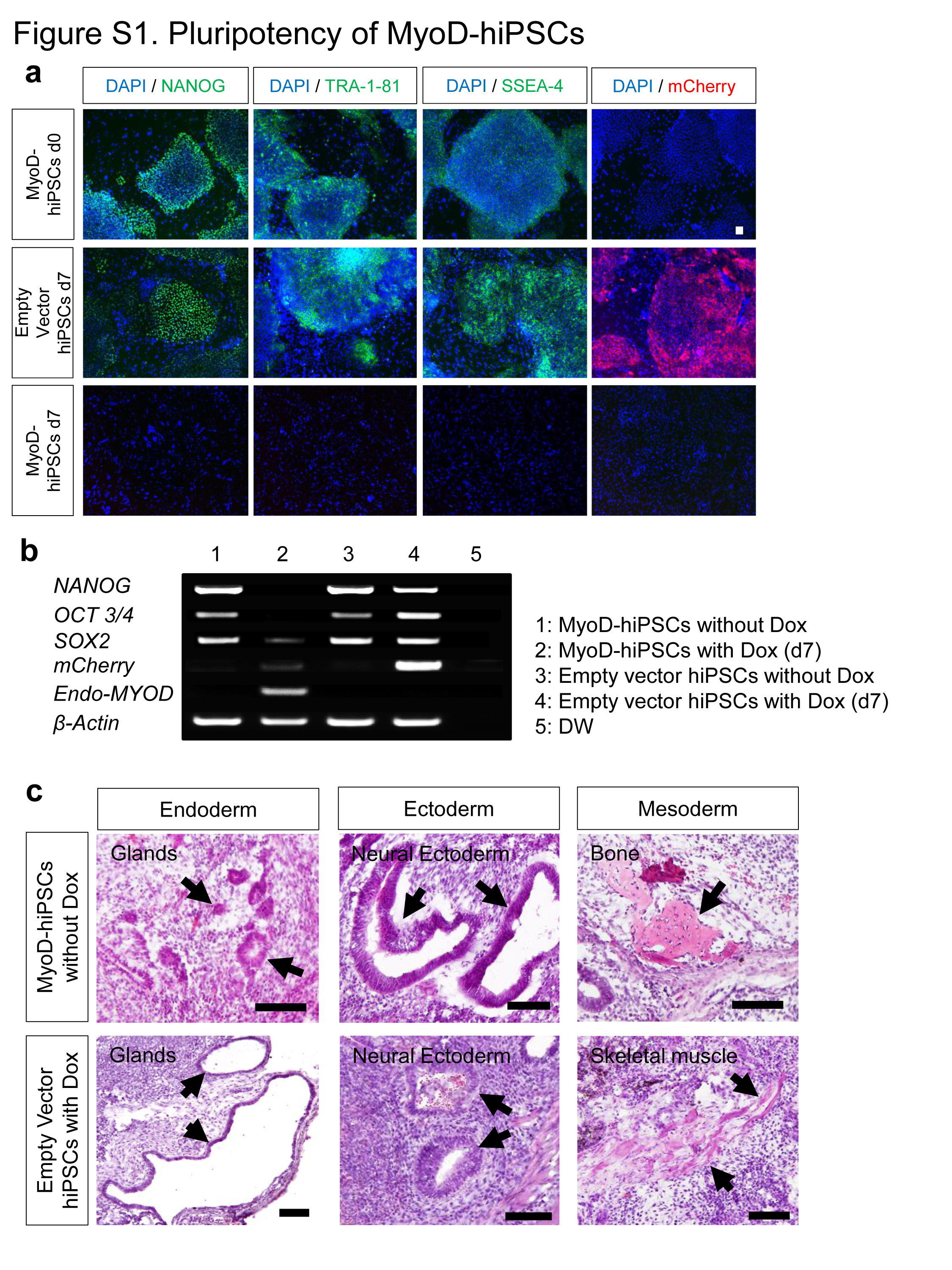

Supplement: Figure S1 — Evaluation of pluripotency of MyoD-hiPSCs. (a) Immunohistochemistry of undifferentiated markers. Scale bar = 100 µm. (b) RT-PCR analysis for undifferentiated markers. (c) Teratoma formation assay from MyoD-hiPSCs and empty vector transduced hiPSCs. H&E staining of teratoma formed in TA muscle from NOD/scid mouse. Three germ layers formed in teratoma were shown in each panel, respectively. Arrows indicate each germ layer, respectively. Scale bars = 100 µm. (TIF) [file pone.0061540.s001.tif]

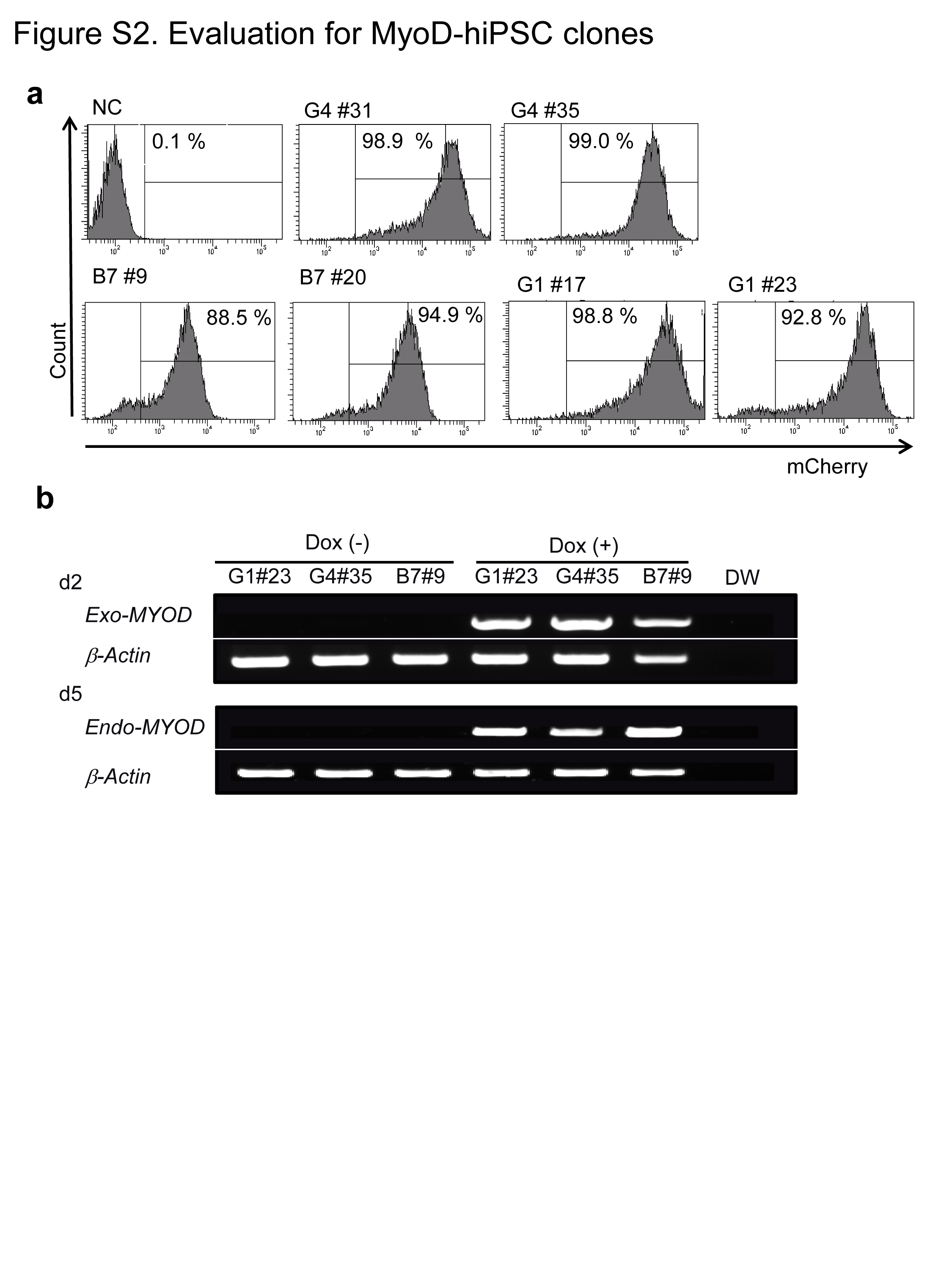

Supplement: Figure S2 — Evaluation for MyoD-hiPSC clones. (a) Expression of mCherry which is synonymous with exogenous MyoD1 driven by Dox treatment for 24 h. (b) RT-PCR analyses of MyoD-hiPSC clones. Cloned MyoD-hiPSCs had no leaky expression of exogenous MyoD1 without Dox, while they could express exogenous MyoD1 24 h after Dox addition. Endogenous MYOD1 could be promoted 96 h after Dox addition. (TIF) [file pone.0061540.s002.tif]

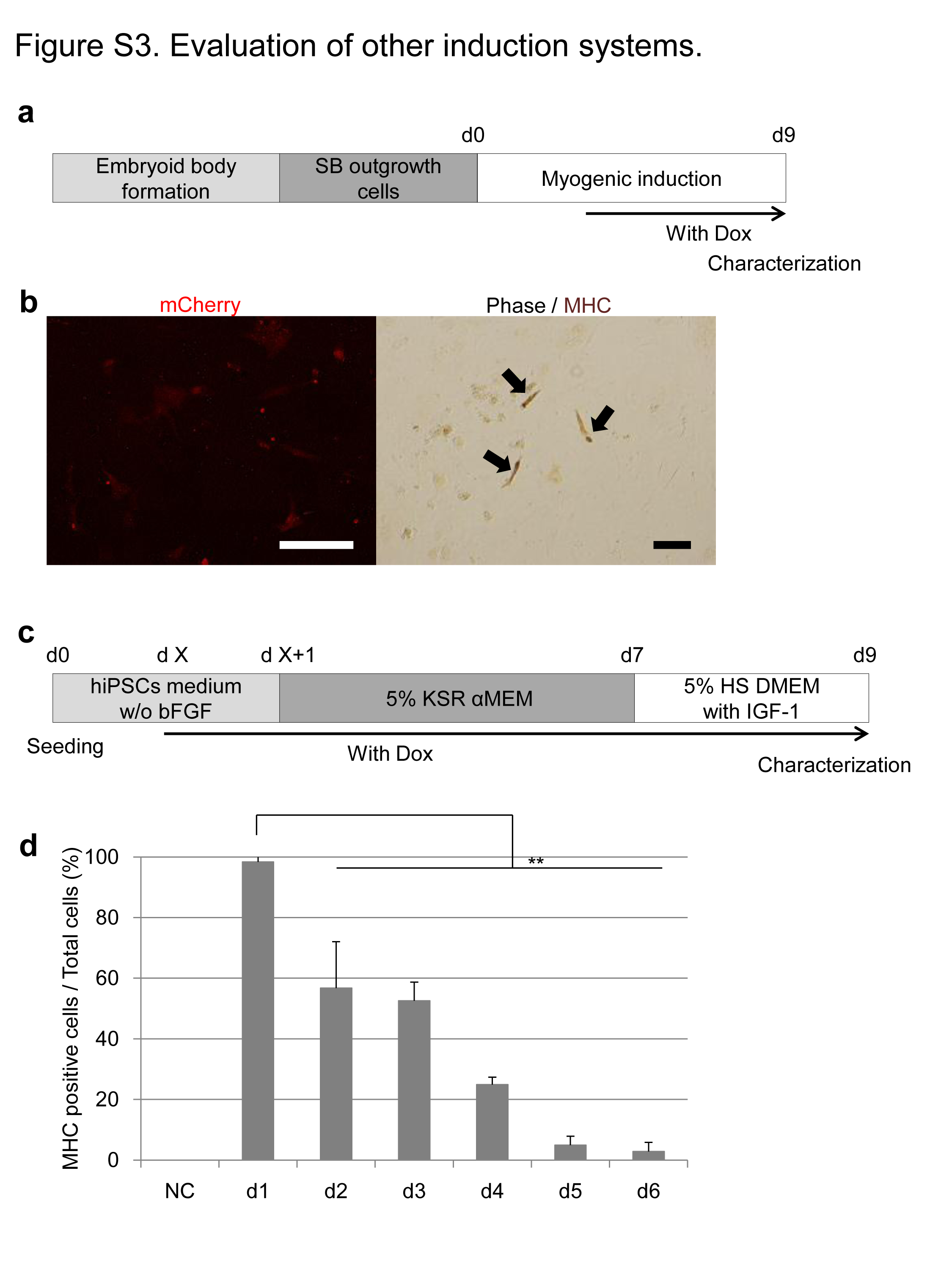

Supplement: Figure S3 — Other myogenic induction methods by SB-OGs system or changing Dox-addition days. (a) Protocol of myogenic induction via EB outgrowth. (b) Expression of mCherry and immunohistochemistry of MHC. Scale bars = 100 µm. (c) Protocol of changing the timing of dox-addition. (d) The percentage of MHC positive cells per total cells. **p<0.01. (TIF) [file pone.0061540.s003.tif]

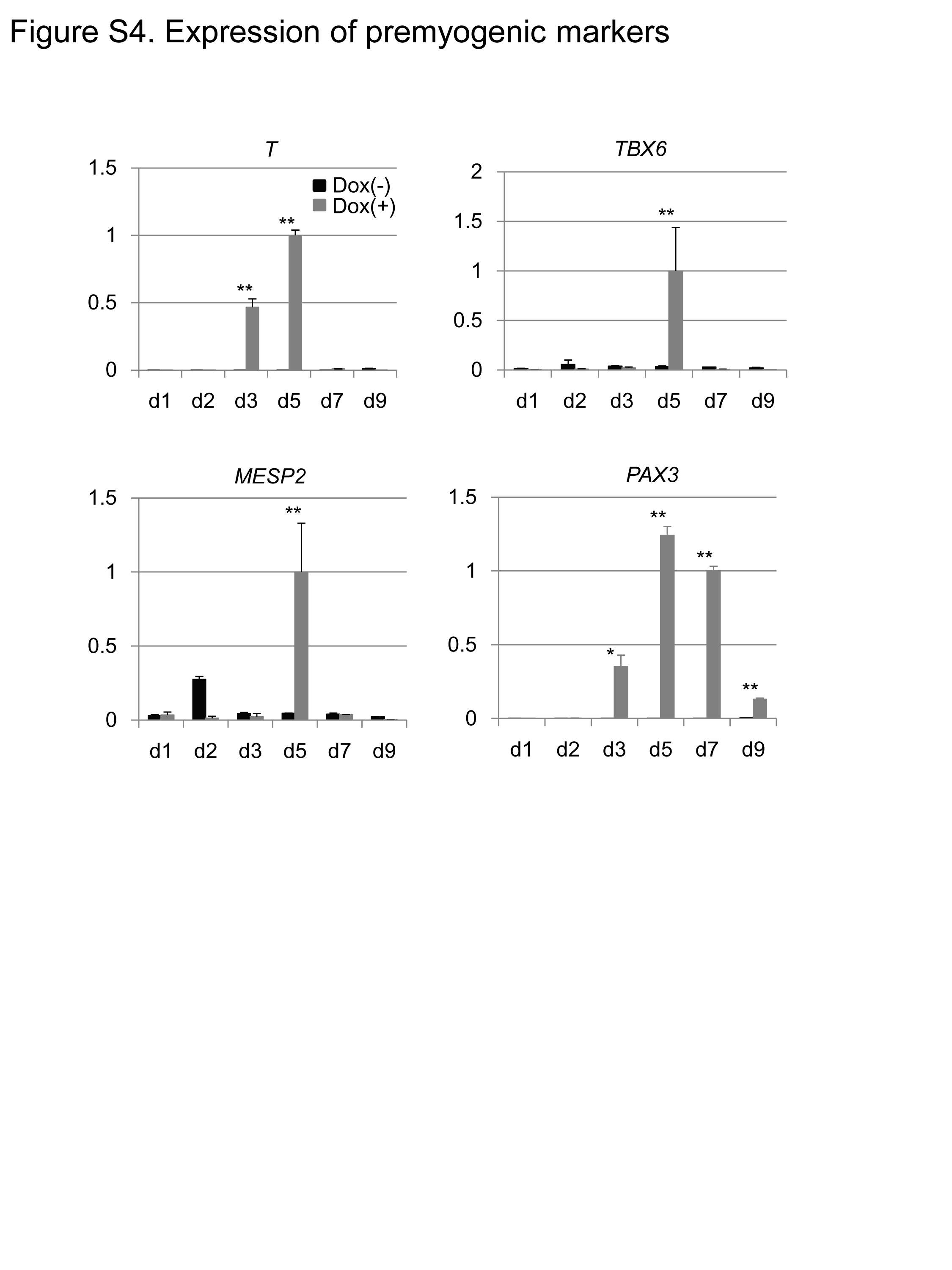

Supplement: Figure S4 — Expression of premyogenic mesodermal markers. Quantitative real time PCR for premyogenic markers was performed during MyoD-hiPSC differentiation in B7 #9 MyoD-hiPSC clone with (gray bars) or without (black bars) Dox administration (n = 3). Data are shown as the mean ± SD. The data were standardized by β-actin using embryoid body. The data on d5 or d7 = 1. *p<0.05, **p<0.01, respectively. (TIF) [file pone.0061540.s004.tif]

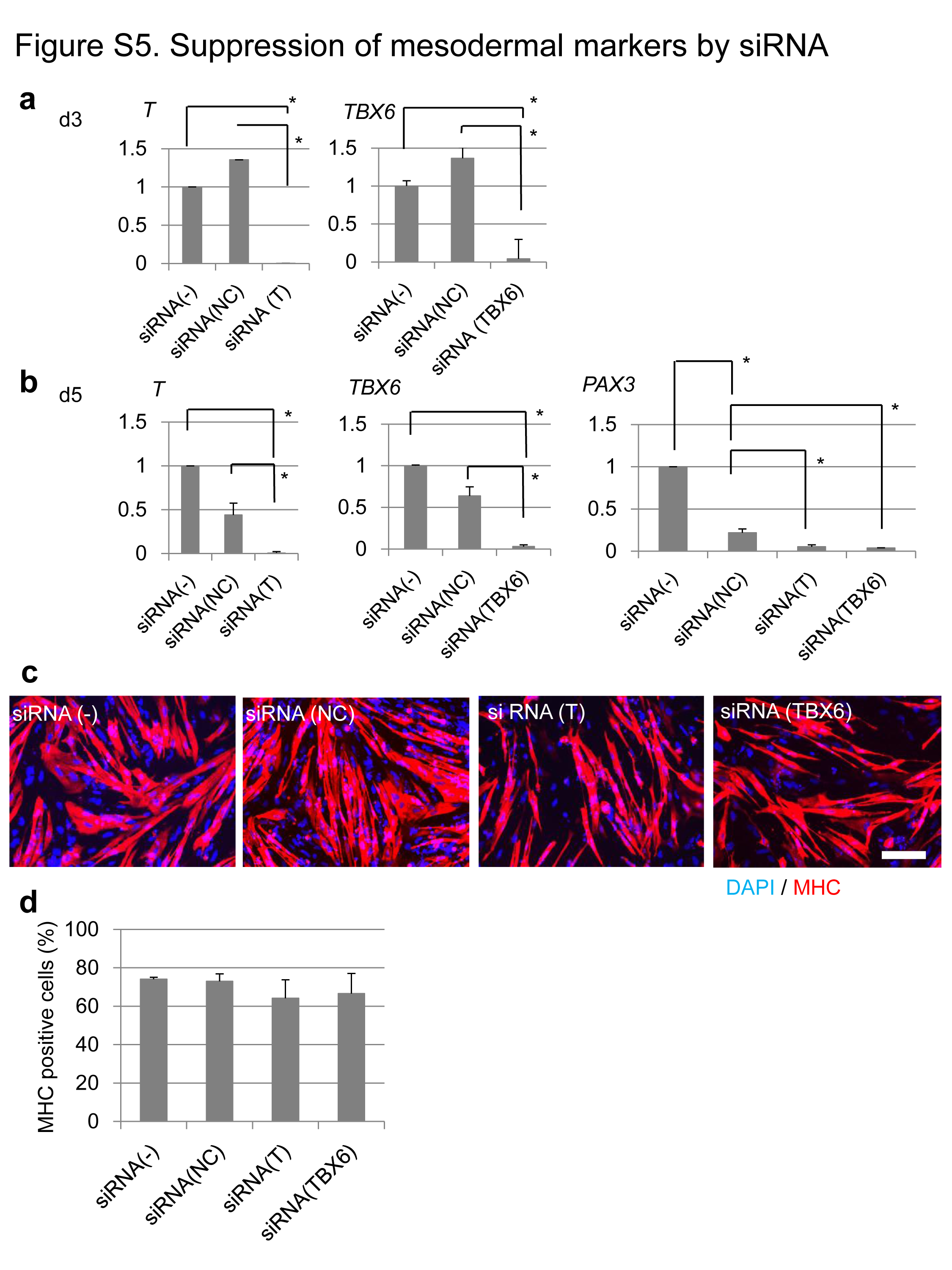

Supplement: Figure S5 — Suppression of mesodermal markers by siRNA. The siRNA reagent targeting T or TBX6 was added to differentiation culture of MyoD-hiPSCs (clone B7 #9) at d0 or d3 (a, b). Quantitative real time PCR was then performed on d3 (a) or d5 (b) (n = 3). The expression of PAX3 was suppressed by siRNAs for both T and TBX6. *p<0.05 (c) The expression of MHC in differentiated MyoD-hiPSCs with or without siRNA treatment on d9. Scale bar = 100 µm. (d) Percentage of MHC positive cells 9 days after differentiation with or without siRNA treatment in B7 #9 MyoD-hiPSC clone (n = 3). (TIF) [file pone.0061540.s005.tif]

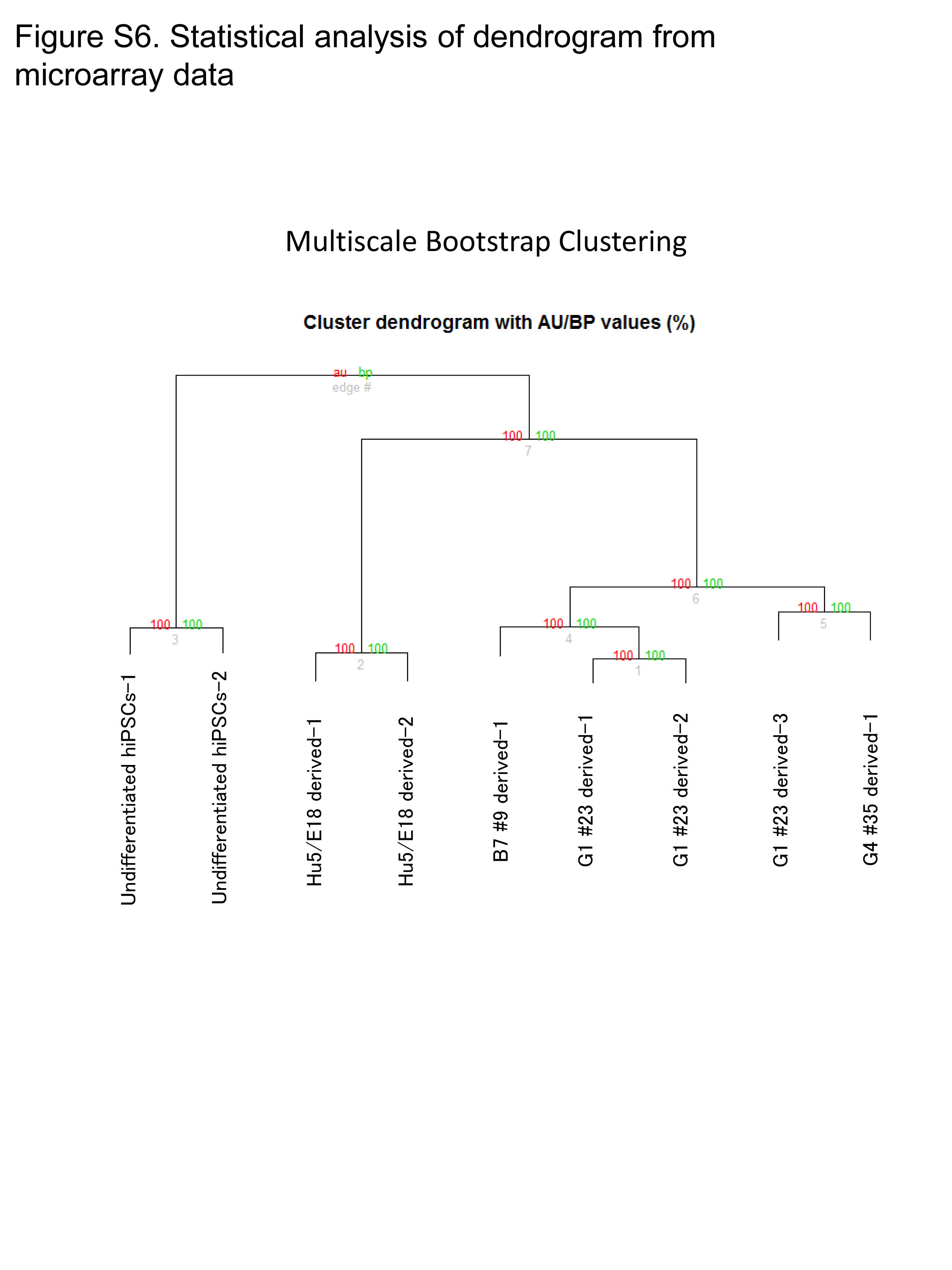

Supplement: Figure S6 — Multiscale bootstrap clustering for the data from microarray. The number of repeated calculation was 1000 times. Abbreviated words “AU” and “BP” means “apporoximately unbiased p-value” and “bootstrap probability,” respectively. Distance means correlation. Cluster method was average. (TIF) [file pone.0061540.s006.tif]

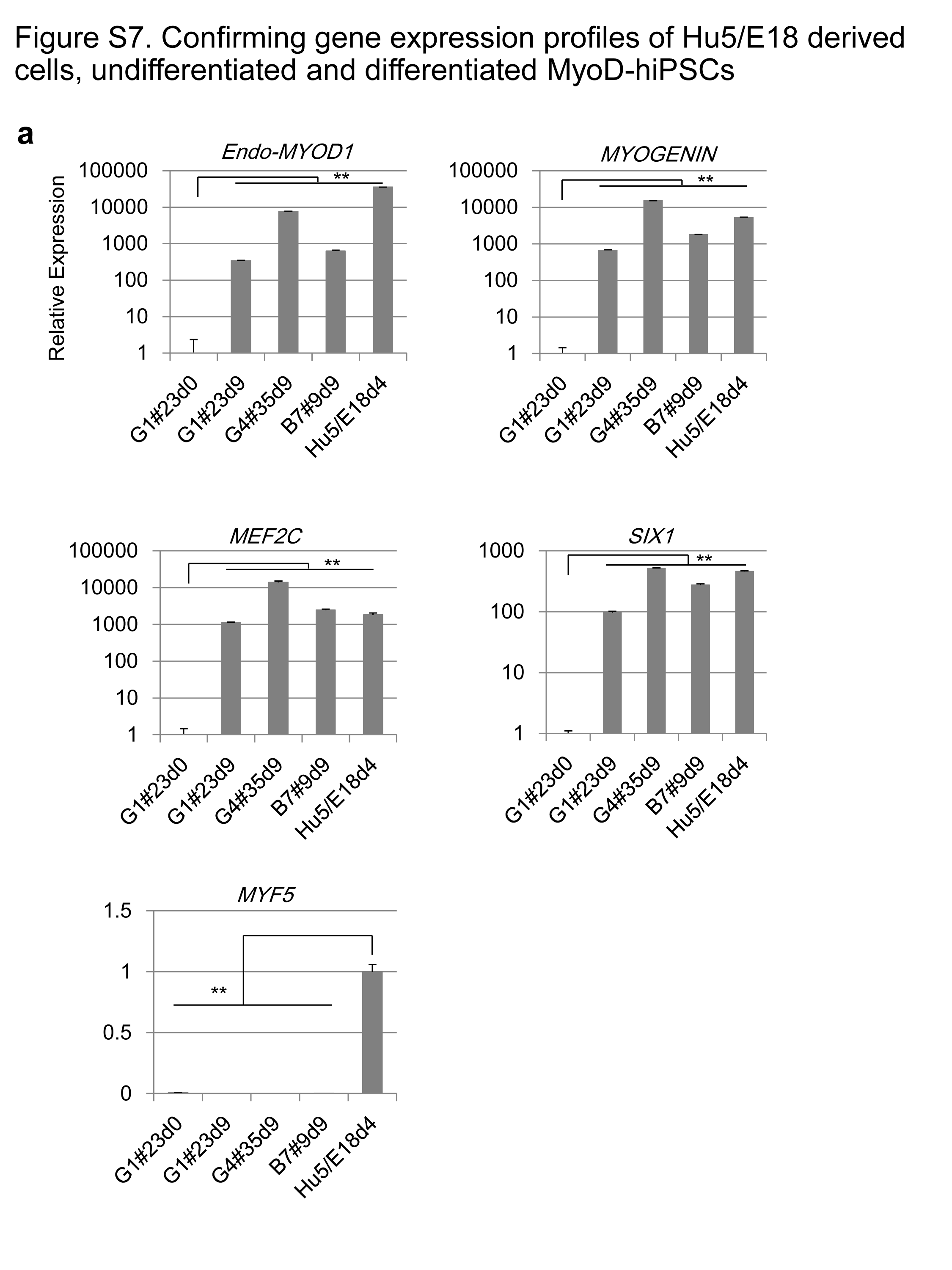

Supplement: Figure S7 — Confirmation of the data obtained from microarray by quantitative real time PCR. (a) Relative gene expression of transcription factors which are significant in microarray analyses. Data are listed as mean ± S.D. The data were standardized by β-actin using teratoma. The data on d0 = 1. The data in Endo-MYOD1, MYOGENIN, MEF2C and SIX1 were expressed with logarithmic Y axes because differentiated cells showed extremely high values, respectively. **p<0.01. (TIF) [file pone.0061540.s007.tif]

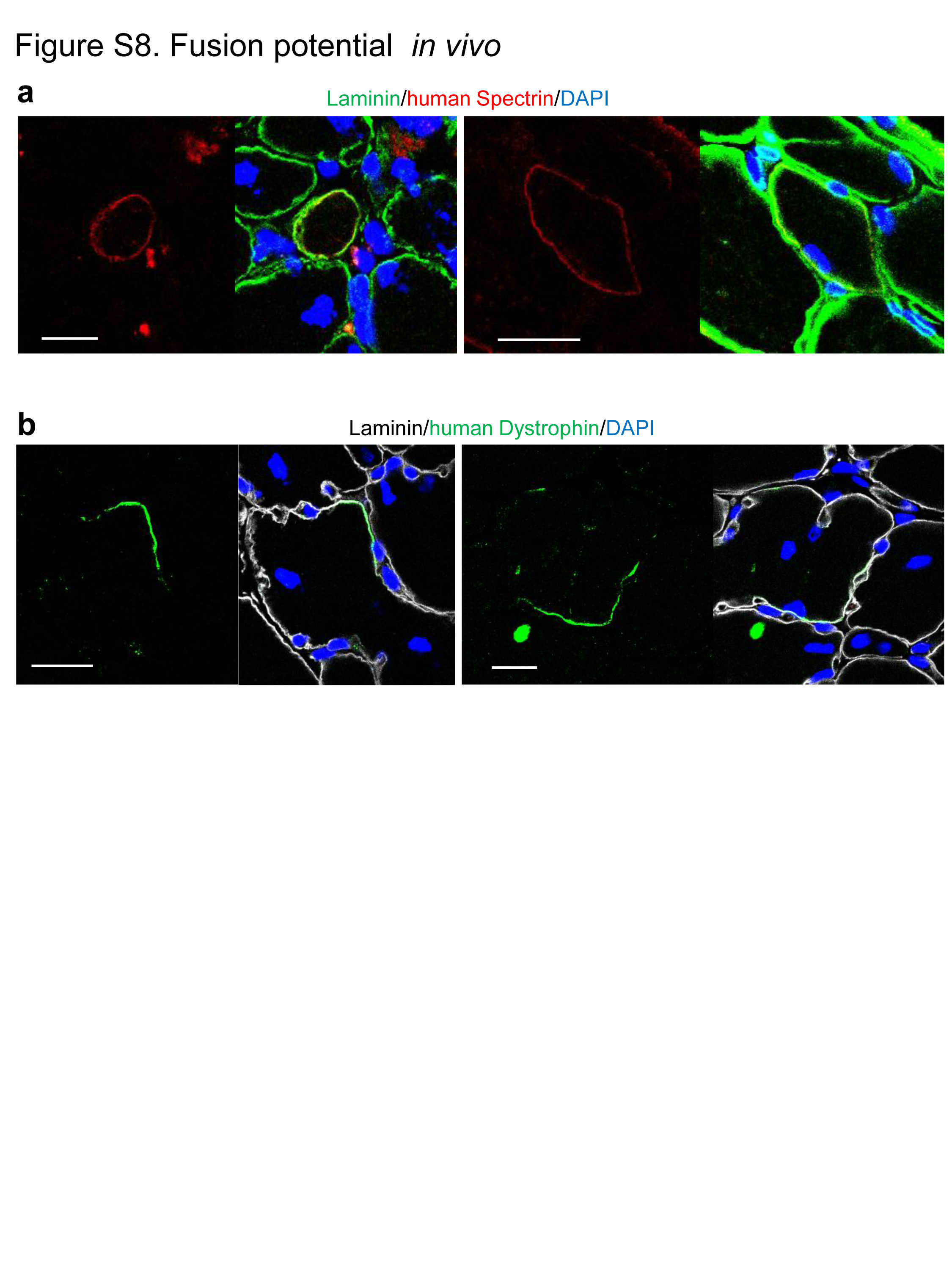

Supplement: Figure S8 — Fusion potential in vivo. Immunohistochemistry of TA muscles from NOD/Scid-DMD mice after 28 days after transplantation of d6 MyoD-hiPSCs. Scale bars = 20 µm. (a) Human Spectrin expression (red) was detected along with Laminin (green). (b) Human DYSTROPHIN expression (green) was detected along with Laminin (white). (TIF) [file pone.0061540.s008.tif]

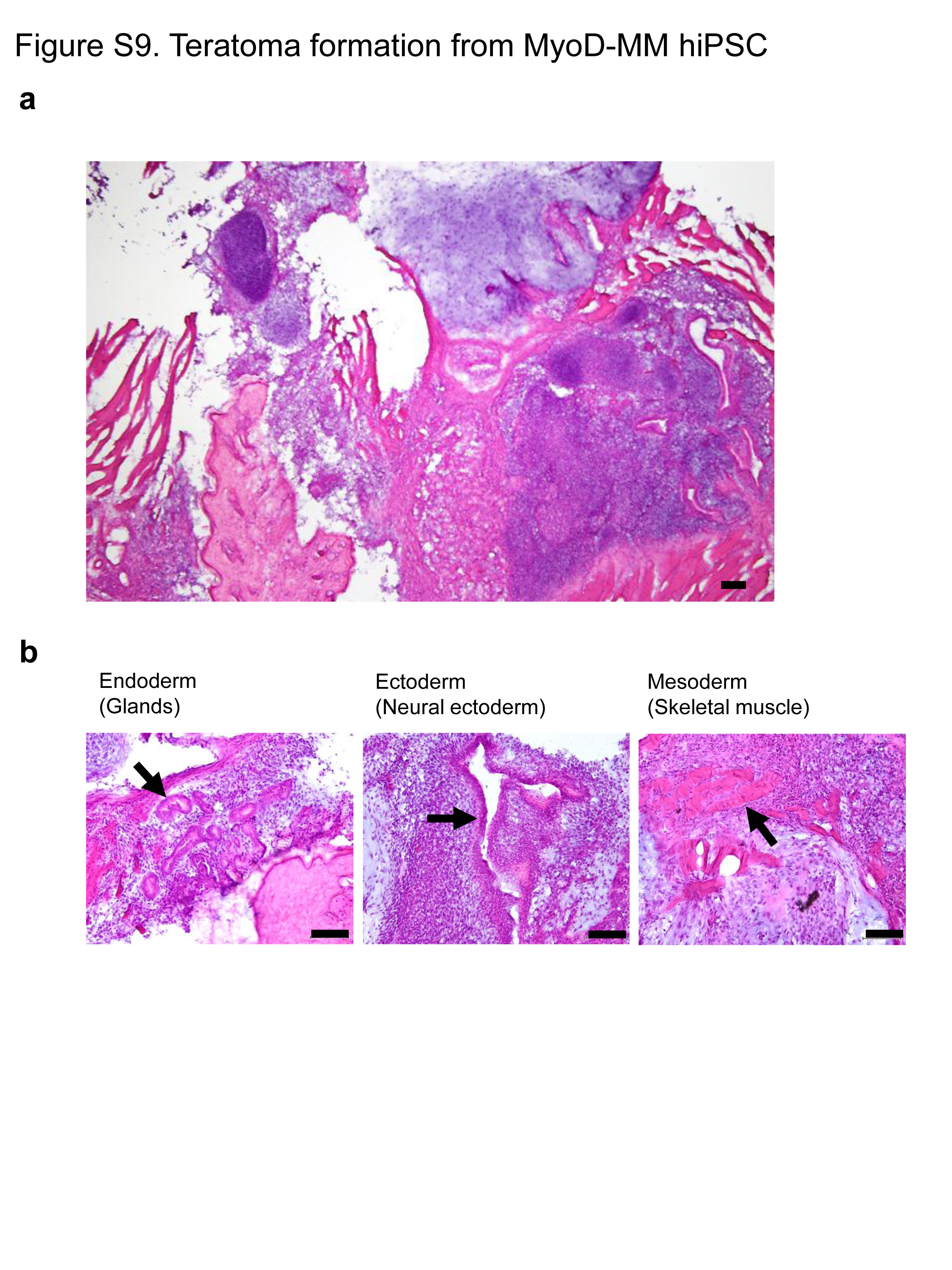

Supplement: Figure S9 — Teratoma formation assay from MyoD-MM hiPSCs. (a) H&E staining of teratoma formed in TA muscle from NOD/scid mouse. Scale bar = 100 µm. (b) H&E staining of three germ layers formed in teratoma. Arrows indicate each germ layer, respectively. Scale bars = 100 µm. (TIF) [file pone.0061540.s009.tif]
