# Supplementary material for: Efficient and Reproducible Myogenic Differentiation from Human iPS Cells: Prospects for Modeling Miyoshi Myopathy In Vitro
Source: PLoS One. 2013 Apr 23;8(4):e61540. doi: 10.1371/journal.pone.0061540 (PMC3633995; doi:10.1371/journal.pone.0061540)
Supplement: Table S1 — PCR-primers were listed for both RT-PCR and quantitative real-time RT-PCR. (DOCX) [file pone.0061540.s010.docx]

**Table S1.** Primes used for RT-PCR

Primers for conventional RT-PCR

| Genes | Sequences |
| --- | --- |
| β-Actin | 5’- CTCTTCCAGCCTTCCTTCCT -3’ |
|  | 5’- CACCTTCACCGTTCCAGTTT -3’ |
| exo-MyoD | 5’- CACCATGGAGCTACTGTCGCCA -3’ |
|  | 5’- TCAGAGCACCTGGTATATCGGGT -3’ |
| endo-MyoD | 5’- GACTGCCAGCACTTTGCTATCT -3’ |
|  | 5’- CCTCAGAGCACCTGGTATATCG -3’ |
| c-Myc | 5’- GCGTCCTGGGAAGGGAGATCCGGAGC -3’ |
|  | 5’- TTGAGGGGCATCGTCGCGGGAGGCTG -3’ |
| Oct 3/4 | 5’- GACAGGGGGAGGGGAGGAGCTAGG -3’ |
|  | 5’- CTTCCCTCCAACCAGTTGCCCCAAAC -3’ |
| Nanog | 5’- CAGCCCCGATTCTTCCACCAGTCCC -3’ |
|  | 5’- CGGAAGATTCCCAGTCGGGTTCACC -3’ |
| Sox2 | 5’- GGGAAATGGGAGGGGTGCAAAAGAGG -3’ |
|  | 5’- TTGCGTGAGTGTGGATGGGATTGGTG -3’ |
| SeV | 5’- GGATCACTAGGTGATATCGAGC -3’ |
|  | 5’- ACCAGACAAGAGTTTAAGAGATATGTATC -3’ |
| mCherry | 5’- CATCCCCGACTACTTGAAGC -3’ |
|  | 5’- CCCATGGTCTTCTTCTGCAT -3’ |

Primers for Quantitative real-time RT-PCR

| Genes | Sequences |
| --- | --- |
| β-Actin | 5’- CACCATTGGCAATGAGCGGTTC -3’ |
|  | 5’- AGGTCTTTGCGGATGTCCACGT -3’ |
| mCherry | 5’- CATCCCCGACTACTTGAAGC -3’ |
|  | 5’- CCCATGGTCTTCTTCTGCAT -3’ |
| endo-MyoD | 5’- CACTCCGGTCCCAAATGTAG -3’ |
|  | 5’- TTCCCTGTAGCACCACACAC -3’ |
| CK-M | 5’- ACATGGCCAAGGTACTGACC -3’ |
|  | 5’- TGATGGGGTCAAAGAGTTCC -3’ |
| Dystrophin | 5’- GATGCACGAATGGATGACAC -3’ |
|  | 5’- TGTGCTACAGGTGGAGCTTG -3’ |
| Myogenin | 5’- TGGGCGTGTAAGGTGTGTAA -3’ |
|  | 5’- CGATGTACTGGATGGCACTG -3’ |
| Mesp2 | 5’- ACTGCCCCAAGATACAGTCG -3’ |
|  | 5’- ACAGGGCTCTGGAGACACAG -3’ |
| T | 5’- ACCCAGTTCATAGCGGTGAC -3’ |
|  | 5’- CATTGGGAGTACCCAGGTTG -3’ |
| Tbx6 | 5’- AGCCTGTGTCTTTCCATCGT -3’ |
|  | 5’- AGGCTGTCACGGAGATGAAT -3’ |
| Pax3 | 5’- AGGAAGGAGGCAGAGGAAAG -3’ |
|  | 5’- CAGCTGTTCTGCTGTGAAGG -3’ |
| Oct 3/4 | 5’- GACAGGGGGAGGGGAGGAGCT AGG -3’ |
|  | 5’- CTT CCC TCC AAC CAG TTG CCC CAA AC -3’ |
| Nanog | 5’- CAGCCCCGATTCTTCCACCAGTCCC -3’ |
|  | 5’- CGGAAGATTCCCAGTCGGGTTCACC -3’ |
| Sox2 | 5’- GGGAAATGGGAGGGGTGCAAA AGAGG -3’ |
|  | 5’- TTGCGTGAGTGTGGATGGGATTGGTG -3’ |
| Myf5 | 5’- tcacctcctcagagcaacct -3’ |
|  | 5’- attaggccctcctggaagaa-3’ |
| MEF2C | 5’- cgagatacccacaacacacg -3’ |
|  | 5’- cgcttgactgagggactttc -3’ |
| Six1 | 5’- agttctcgcctcacaaccac -3’ |
|  | 5’- acacccctcgacttctcctt -3’ |
